# Ecosystem metabolism and nitrogen budget of a glacial Fjord in the Arctic

**DOI:** 10.1038/s41598-025-06953-3

**Published:** 2025-07-02

**Authors:** Pedro Duarte, Laura Castro de la Guardia, Philipp Assmy, Anette Wold, Agneta Fransson, Melissa Chierici, Allison Bailey, Andrew Hodson, Andreas Alexander, Catarina Magalhães, Geir Wing Gabrielsen, Jon Albretsen, Lukas Frank, Sarat Chandra Tripathy, Carlos Smerdou, Francisco J.L. Gordillo, Pablo Cobos, David Velázquez, Peter Convey, Francesco De Rovere, Haakon Hop

**Affiliations:** 1 Norwegian Polar Institute, Fram Centre, Tromsø, 9296 Norway; 2 Present Address: Scottish Association for Marine Science (SAMS) Dunstaffnage Marine Laboratory, Oban, PA37 1QA UK; 3 Institute of Marine Research, Tromsø, 9007 Norway; 4 University Centre in Svalbard, Longyearbyen, 9171 Norway; 5 Department of Civil Engineering and Environmental Sciences, Western Norway University of Applied Sciences, Sogndal Campus, Bergen, 5020 Norway; 6 Norwegian Water Resources and Energy Directorate, Oslo, 0301 Norway; 7 Interdisciplinary Centre of Marine and Environmental Research, University of Porto, Terminal de Cruzeiros do Porto de Leixões, Av. General Norton de Matos s/n, Porto, 4450-208 Portugal; 8 Institute of Marine Research, PO Box 1870, Bergen, 5817 Norway; 9 University of Bergen, PO Box 7800, Bergen, 5020 Norway; 10 National Centre for Polar and Ocean Research (NCPOR), Vasco-da-Gama, Goa, 403804 India; 11 Faculty of Sciences, University of Málaga, Málaga, 29071 Spain; 12 Dept. of Biology, Universidad Autonoma de Madrid, Madrid, 28049 Spain; 13 British Antarctic Survey, NERC, Cambridge, UK; 14 Department of Zoology, University of Johannesburg, Auckland Park, 2006 South Africa; 15 Department of Environmental Sciences, Informatics and Statistics, Ca’ Foscari University of Venice, Venezia, Italy; 16 Consiglio Nazionale delle Ricerche, Istituto di Scienze Polari (CNR-ISP), Bologna, Italy; 17 Faculty of Sciences, University of Porto, 4169-007 , Rua do Campo Alegre, s/n, Porto, Portugal

**Keywords:** Net ecosystem metabolism, Glacial fjord, Nutrient sink/source, Marine endmembers, Freshwater endmembers, Kongsfjorden, Biogeochemistry, Ecology, Environmental sciences, Ocean sciences, Physics

## Abstract

Fjords in the Arctic are changing rapidly due to multiple factors including increasing air temperatures, the influx of Atlantic Water (Atlantification), sea-ice loss, retreat of tidewater glaciers, increased freshwater discharges, pollution and tourism. Understanding how these changes affect ecosystem processes and functions and, thus, services to society is critical. Net Ecosystem Metabolism (NEM) offers a holistic measure of ecosystem functioning and services, reflecting the balance between autotrophic and heterotrophic processes and the sink/source role of an ecosystem for nutrients and carbon. Using a 10-year dataset we quantify the main nutrient sources and sinks in Kongsfjorden (Svalbard) and estimate NEM using a method based on mixing diagrams combined with an ocean circulation model. We show that Kongsfjorden is a nutrient and carbon sink primarily supported by nutrient inputs from the adjacent shelf sea with terrestrial run-off playing a secondary role. Given the ongoing changes in the Arctic, driven by global warming and its associated effects, we recommend monitoring NEM as an integrated measure of the state of coastal ecosystems, considering the disproportionately large role of coastal regions in the global carbon budget.

**Supplementary Information:**

The online version contains supplementary material available at 10.1038/s41598-025-06953-3.

## Introduction

Coastal ecosystems are among the most productive on the planet^1^. However, large uncertainties remain about the magnitude of carbon and nutrient fluxes in these systems [for example^2^]. The Arctic is changing rapidly due to global warming, accompanied by shifts from traditional economic activities towards tourism and other industrial activities [for example^3^]. Therefore, it is important to monitor Arctic fjords in line with lower latitudes [for example^4^]. Changes in nutrient and carbon budgets are anticipated due to thawing sea ice, permafrost, glaciers and ice sheets [for examples^5,6^], amongst other possible causes. Such changes will have implications for ecosystem processes and functions and, thus, services to society, such as Arctic wildlife, harvestable resources, and the role of coastal ecosystems as sinks/sources of greenhouse gases and nutrients, further reinforcing the monitoring needs.

Net Ecosystem Metabolism (NEM) is a holistic measure that informs about overall ecosystem functioning. It is equivalent to community metabolism computed from the oxygen balance in streams, as described by Odum^7^. NEM reflects the balance between autotrophic and heterotrophic processes computed from the difference between gross primary production and ecosystem respiration^8^. NEM varies widely amongst ecosystems depending on the relative balance between nutrient input (which increases primary production and NEM) and organic carbon loading (which increases heterotrophy and decreases NEM)^9^. Tracking NEM can indicate the effects of stressors such as pollution or climate change on ecosystem functions and it is helpful in monitoring ecosystems^10^providing knowledge and recommendations for management and conservation practices.

NEM can be computed using several methods, as reviewed by Staehr et al. ^11^. These methods include (i) bottle and chamber incubations, (ii) open water methods based on Lagrangian or Eulerian sampling, (iii) nutrient budgets and (iv) scaling relationships with the area or the volume of coastal ecosystems^2^. Methods (i) and (ii) are based on direct measurements of dissolved oxygen or dissolved inorganic carbon concentration changes over periods of hours to days, while method (iii) is based on mass balances of physical and chemical inputs and outputs (e.g. water and nutrients) to/from a given ecosystem at seasonal and annual time scales. The spatial and temporal variability in coastal ecosystems complicates upscaling of methods (i) and (ii), while nutrient budgets (iii) such as the Land Ocean Interaction Along the Coastal Zone (LOICZ) approach, often focused on nitrogen or phosphorus^8,10,12,13^ are more data intensive and challenging to apply in fjords, which have distinct vertical layers of surface, intermediate and bottom waters [for example^14^].

**Fig. 1:**
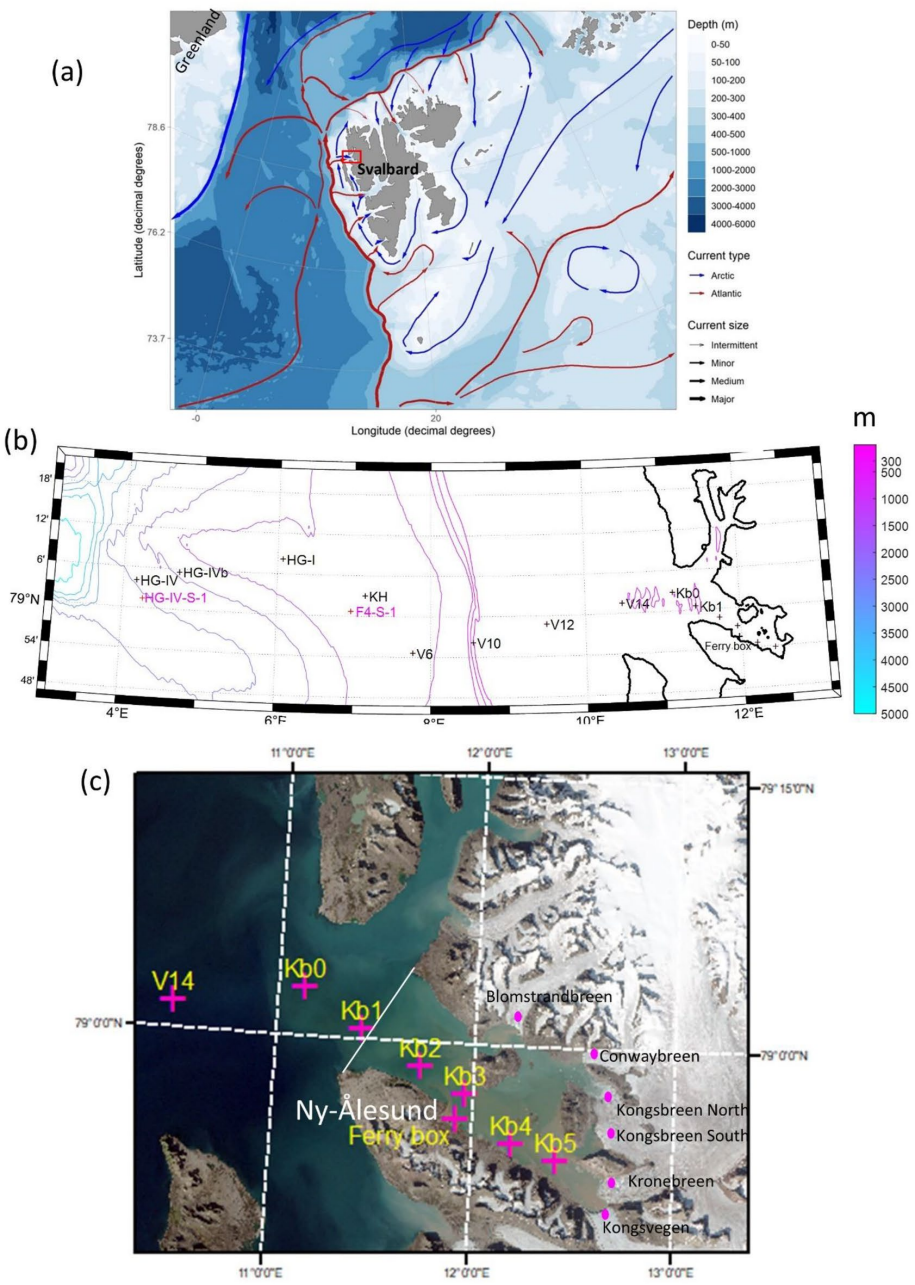
(**a**) Geographical context of Kongsfjorden (red square), located on West Spitsbergen in Svalbard. The West Spitsbergen Current is shown in red and the Spitsbergen Polar Current in blue. This panel was produced with the PlotSvalbard R package^17^ (https://github.com/MikkoVihtakari/PlotSvalbard?tab=GPL-2.0-1-ov-file). (**b**) Location of sampling sites from the inner Kongsfjorden, across the shelf and into the Fram Strait. All stations with black crosses (except the Ferry Box of the AWIPEV underwater observatory, located in front of Ny-Ålesund) are part of the MOSJ and AMUST datasets. Stations with magenta crosses are part of the Torres-Valdes et al. dataset^18^ (refer to Table S1). The bathymetry represented by the contour lines combines the K160_bgc model bathymetry and the International Bathymetric Chart of the Arctic Ocean downloaded from https://www.gebco.net/data_and_products/gridded_bathymetry_data/arctic_ocean/at 200 m resolution; (**c**) General view of Kongsfjorden and the terrestrial and glacial surroundings. Also included are the locations of MOSJ sampling sites, Ny-Ålesund and the Ferry Box in Kongsfjorden (magenta crosses, with the same cross used to show the location of Ny-Ålesund and the Ferry Box), and the location of the tidewater glacier fronts (magenta dots), using data from the Norwegian Polar Institute^19^ over an aerial image from Copernicus Sentinel Data. The white line at the mouth of Kongsfjorden delimits the fjord area, which is the focus of this study to calculate nutrient budgets. Panels (b, c) were produced/modified using Matlab R2024b.

We hypothesize that it is possible to investigate the nutrient and carbon sink/source role of fjords using “traditional” monitoring data, based on snapshots of environmental variables, if we can differentiate concentration changes due to mixing, based on salinity, from biogeochemical processes, based on non-conservative variables, such as nitrogen or dissolved inorganic carbon (DIC). We tested this hypothesis for Kongsfjorden, a glacial fjord in Svalbard (Fig. 1).

Long-term monitoring of the atmosphere, glaciers, marine and terrestrial ecosystems coupled with the multidisciplinary knowledge of Kongsfjorden and its adjacent shelf sea, provide a unique multivariate dataset not commonly available elsewhere in the Arctic [for example^15^]. Kongsfjorden may be seen as a harbinger of change, and knowledge about its response to change can help to anticipate future modifications in other Arctic coastal systems^16^. Despite research efforts in Kongsfjorden, ecosystem-level measures of state, such as the relative importance of autotrophic *versus* heterotrophic processes and the sink/source role of Kongsfjorden for carbon, nitrogen and other important elements, are still lacking.

The objectives of this study are, therefore, to: (i) synthesize a nitrogen budget for Kongsfjorden to gain insight into the relative importance of various exchange processes; (ii) quantify the role of Kongsfjorden as a sink or source for nitrogen, phosphorus, silicic acid and DIC; and (iii) estimate Konsgfjorden’s NEM. We combine published data for (i) and we use a methodology for (ii) and (iii) based on the analysis of salinity, nutrient and DIC concentration gradients in endmember water masses between Kongsfjorden and the adjacent shelf, combined with estimates of fjord flushing time (FT), obtained using an ocean model. This approach, with relatively low data requirements, has the potential for application in other coastal ecosystems to quantify the relative roles of autotrophy and heterotrophy.

## Results

### Nitrogen budget

**Fig. 2:**
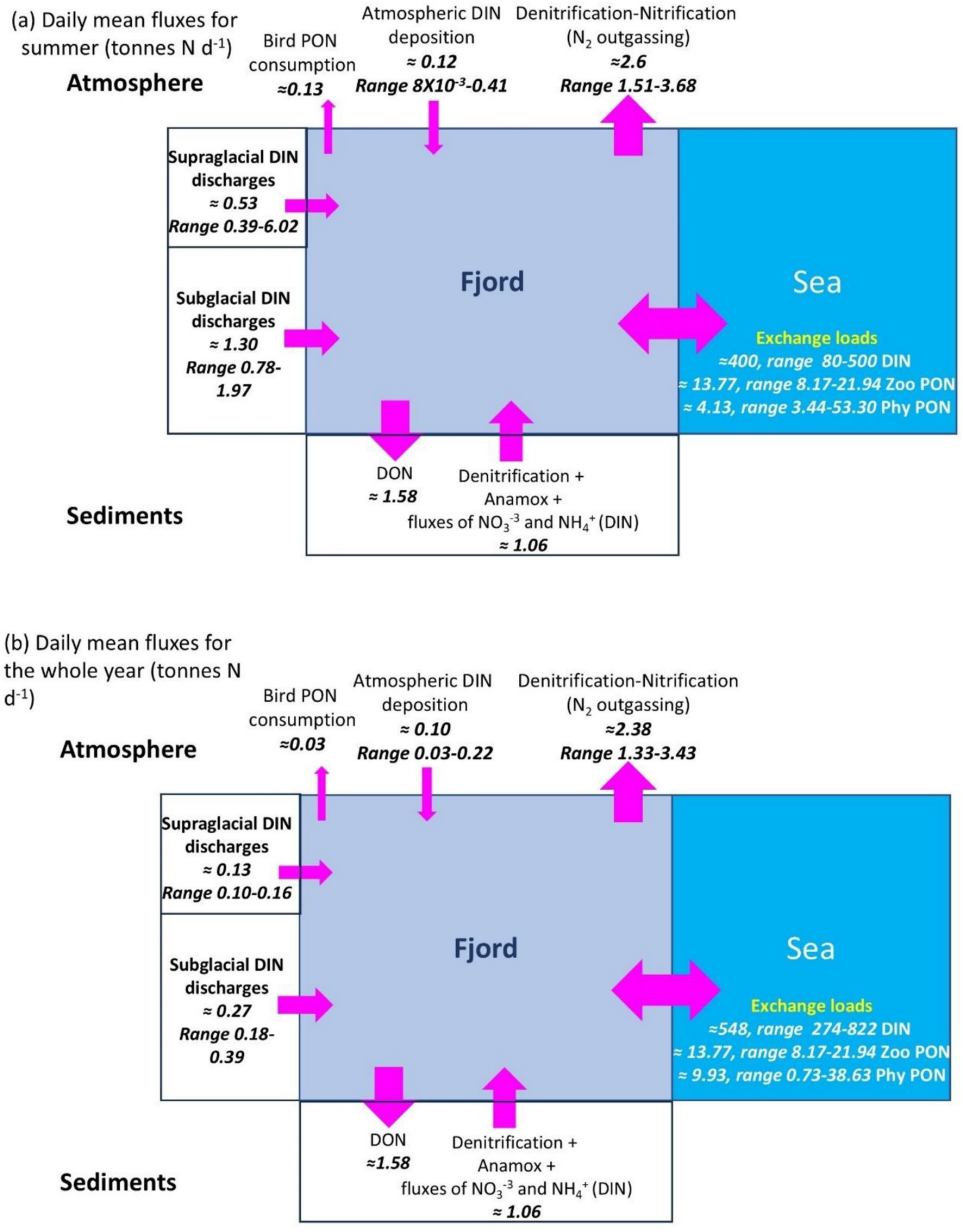
Daily average nitrogen fluxes (tonnes N d^−1^) and ranges (when available) calculated for the period 2011–2023 (depending on data availability), except water-sediment exchanges that are based on^21^ (see text). Fluxes are calculated for (**a**) summer and (**b**) the whole year and for the fjord area limited by the white line in Fig. 1c. Values correspond to dissolved inorganic nitrogen (DIN = nitrate + nitrite + ammonia), N_2_, dissolved organic nitrogen (DON), and particulate organic nitrogen (PON) from phytoplankton (Phy) and zooplankton (Zoo) or seabird prey as specified in the panels.

We compiled nitrogen budgets obtained from various literature and data sources (see **Methodology**). The daily-mean budget for summer (Fig. 2a) showed that dissolved inorganic nitrogen exchanges between the fjord and the shelf (80–500 t N d^−1^) were 1–3 orders of magnitude larger than all other fluxes, followed by exchange of particulate organic nitrogen in the form of phytoplankton and zooplankton in the range of tens of tonnes per day. Atmospheric exchange represents a nitrogen sink due to denitrification (≈ 3 tonnes N d^−1^), based on^20^ (see Supplementary information, Text S1). Riverine inputs are smaller than the losses to the atmosphere, and bird-food consumption (~ 0.13 tonnes N d^−1^) is one of the smallest fluxes. The water-sediment exchanges based on^21^ suggest that the sediment is a nitrogen sink. The daily-mean budget calculated with data for the whole year (Fig. 2b) shows similar relative magnitudes with small differences driven by strong seasonality on some processes such as river discharge.

## Hydrography and fjord-shelf physical-chemical gradients

**Fig. 3:**
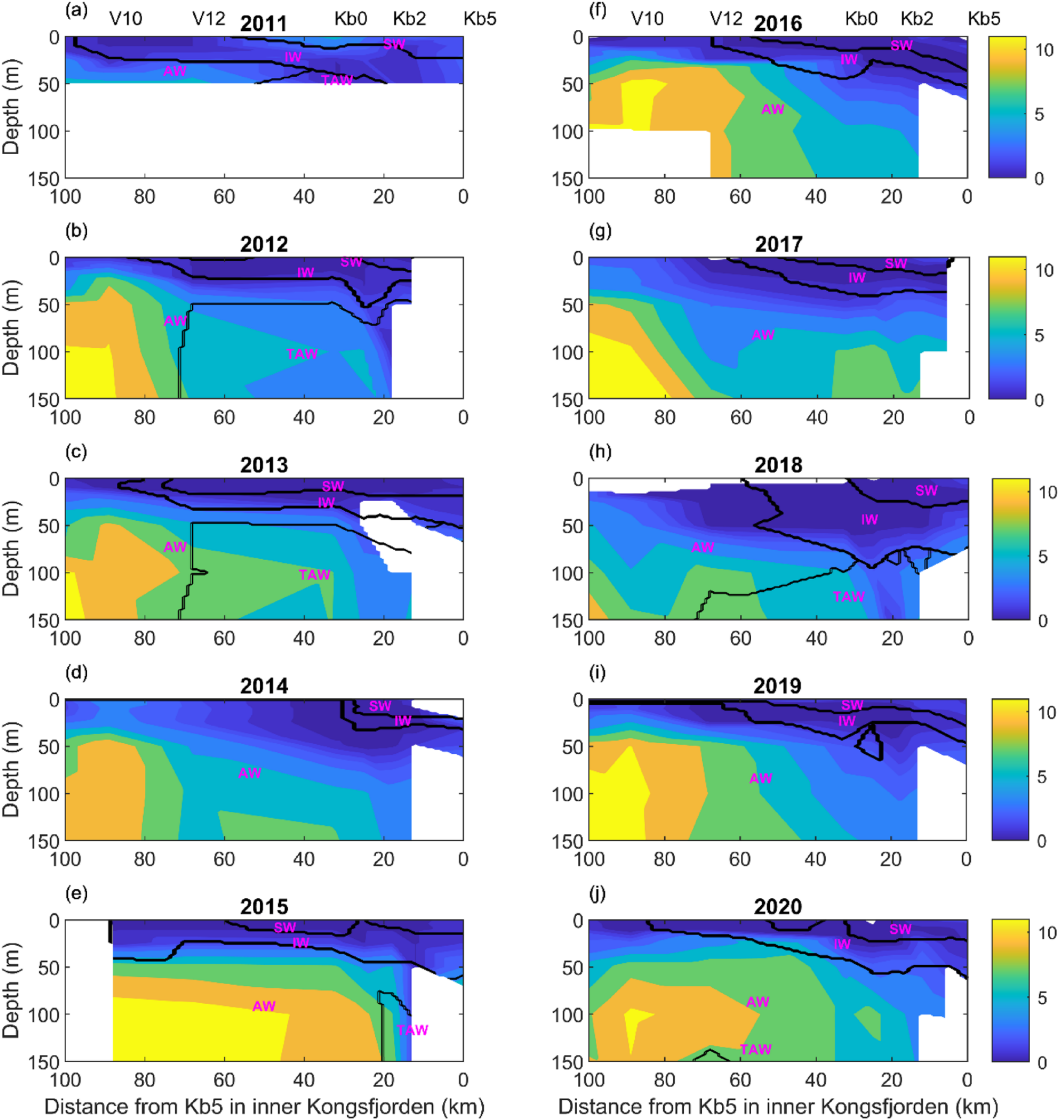
Nitrate + nitrite concentration contours (µmol kg^−1^) for 2011–2020 based on the MOSJ dataset for the Kongsfjorden summer transect (Table S1) and water masses delimited by the black lines, according to Cottier et al.^24^ (AW – Atlantic Water, TAW – Transformed Atlantic Water, SW – Surface Water, and IW – Intermediate Water). A subset of the sampling stations is plotted over panels a, f. Refer to Table S2 for water mass characteristics and Figs. 1b, c for the locations of all sampling stations. The white areas in the contour plots correspond to places where data were not available. In the case of station Kb5 there were no data below 100 m due to depth constraints.

**Fig. 4:**
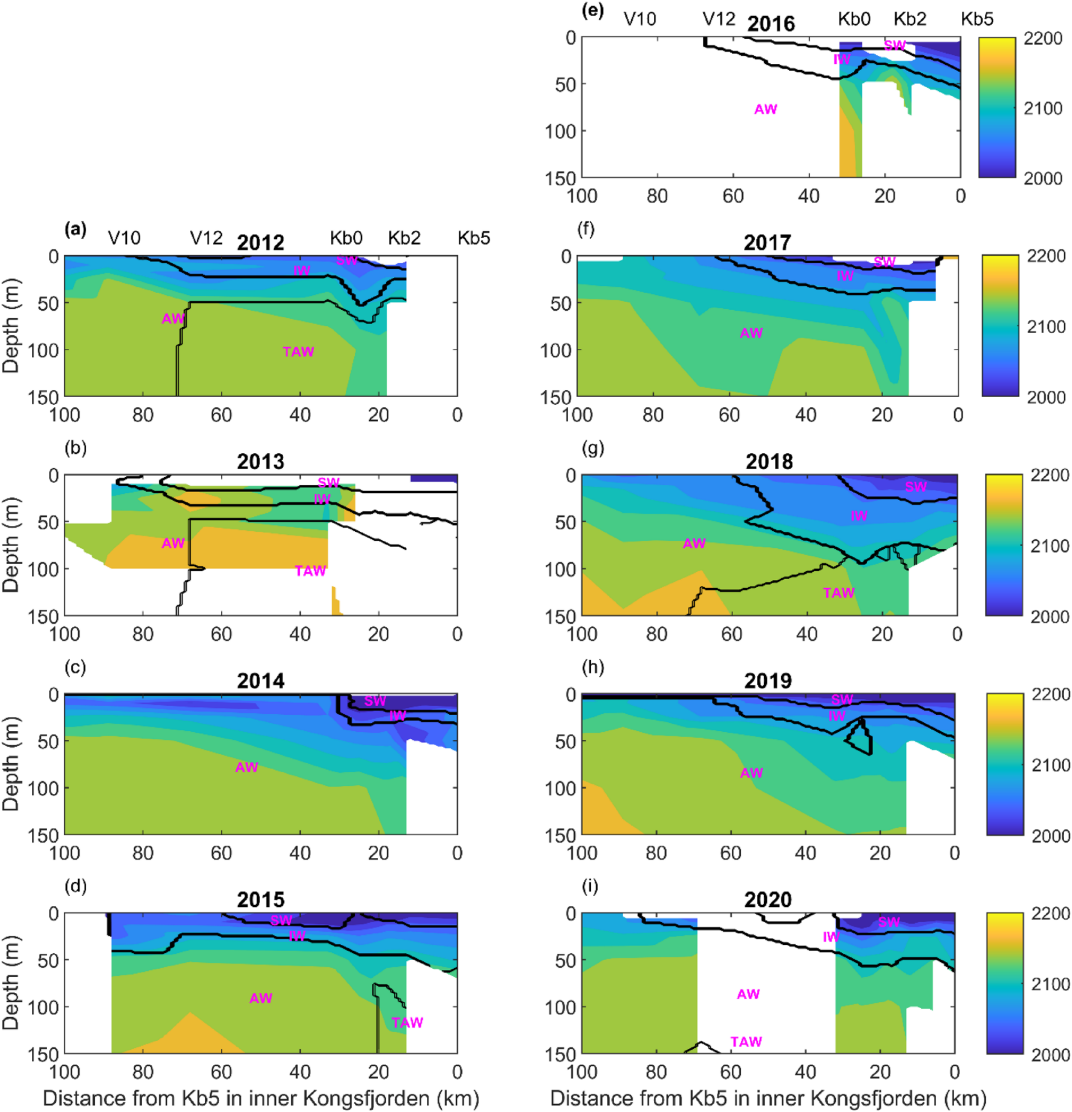
DIC concentration contours (µmol kg^−1^) for the years 2012–2020 based on the MOSJ dataset for the Kongsfjorden summer transect (Table S1) and water masses delimited by the black lines, according to Cottier et al.^24^ (AW – Atlantic Water, TAW – Transformed Atlantic Water, SW – Surface Water, and IW – Intermediate Water). A subset of the sampling stations is plotted over panels a, e. Refer to Table S2 for water mass characteristics and Figs. 1b, c for the locations of all sampling stations. The white areas in the contour plots correspond to places where data were not available. In the case of station Kb5 there were no data below 100 m due to depth constraints.

Four water masses were identified within the top 150 m and along the transect between inner Kongsfjorden and Fram Strait in late July [MOSJ; Ref^22^]: Surface Water (SW), Intermediate Water (IW), Atlantic Water (AW), and Transformed Atlantic Water (TAW) (Figs. 3 and 4, refer to Fig. 1b and c for geographic details and Table S2 for water-mass classification). AW was the dominant water type, followed by IW and SW, except for 2012 and 2013, when AW was followed by TAW. The AW was present at the surface on the shelf and deep Fram Strait (roughly between stations HG-I and V10-12, not shown), while the fjord consistently had a SW layer of 20–30 m thickness, followed by IW down to ~ 50 m depth, and then AW (stations Kb0-Kb5). In 2012, 2013, and 2018, the AW was over a layer of TAW within the fjord area. In Fram Strait, SW and IW were within the top ~ 50 m on some occasions (not shown). However, these water masses may have a different origin than those receiving the same classification but located within the fjord. Data from 2019 to 2020 showed deepening of the lower-salinity surface from the shelf to the fjord and the near-surface thermal stratification (Fig. S7).

Nutrient and DIC concentrations displayed interannual variability, but comparable horizontal and vertical gradients from outer to inner fjord. Summer nitrate + nitrite concentrations were vertically stratified, with a nitracline between ~ 25 and 50 m. The highest concentrations occurred in AW and TAW, with no significant difference between these two water masses (two-tailed *t*-test, *p* > 0.05). Above the nitracline, values decreased to < 1 µmol kg^−1^ in SW, IW and AW. However, on some occasions, there were higher surface values in AW over the shelf, with the shape of the concentration isolines suggesting the occurrence of upwelling (e.g., in 2014, Fig. 3 d). Winter nitrate + nitrite concentrations in surface water were much higher and varied between 9.6 and 11.9 µM^23^. A clear decreasing trend in nitrate + nitrite concentrations was observed from the deep basin into the fjord, within the 0–100 m depth range, correlating with the dilution of AW and its transformation in IW and SW at this depth range within most of the fjord (Fig. 3). Mixing diagrams showed low nitrate + nitrite concentrations in fjord surface water masses (SW and IW) without any clear trend with salinity, whereas deeper water masses showed a positive nitrate + nitrite trend with salinity (Fig. S8).

Dissolved inorganic carbon, phosphate and silicic acid exhibited a similar decreasing gradient from the deep basin to the fjord, whereas no clear patterns were observed for ammonium (Figs. 4, S10, S11, S12 and S14). DIC showed increasing trends with salinity for all water masses (Fig. S9). Phosphate and silicic acid varied with salinity in a similar fashion as nitrate + nitrite (Figs. S13 and S15).

## Biogeochemical Sources-Sinks

Biogeochemical *Sources-Sinks* computed with Eq. 1 (cf. Methodology, **Analysis of nutrients/DIC concentration gradients and quantification of biogeochemical sinks/sources**), revealed that the fjord acted as a summer- sink for nitrate + nitrite in all years (2011–2023), between, on the one hand, SW and IW within the fjord and AW outside the fjord (Table 1). Regarding ammonium, the fjord acted as source but up to one order of magnitude smaller than the nitrate + nitrite sink (Table S6). The fjord also acted as a sink for DIC, phosphate and silicic acid (Table 2, S7 and S8). Adjusting the averaging depth for nitrate + nitrite calculations from 100 m (Table 1) to 50–150 m (Table S9) resulted in less negative fluxes for 50 m and negligible differences for 150 m. We use bold type for the *Sources-Sink* terms listed in Tables 1 and 2 and S6-S8 to indicate the values that were based on significantly different mean concentrations (see **Methodology, Analysis of nutrients/DIC concentration gradients and quantification of biogeochemical sinks/sources**).

The *Sources-Sinks* computed between AW within and outside the fjord also rendered mostly negative values for nitrate + nitrite, phosphorus and silicic acid, even though with smaller differences than for the water-mass comparisons detailed above. However, results were not consistent for DIC, being positive in some years and negative in other years.

Other datasets include nutrient data for other seasons but at a lower horizontal and vertical resolution than that available in the MOSJ dataset (Fig. S16). Available results show that nitrate + nitrite concentrations on the shelf and in Fram Strait were equal or greater than those within the fjord in winter/summer, similar to the results described above and based on summer MOSJ data (Fig. S16), with the fjord seasonality mimicking that observed offshore and nitrate + nitrite concentrations ranging from nearly undetectable in summer to more than 12 µM in winter.

**Table 1:** Biogeochemical nitrate + nitrite *Sources-Sinks* estimated using Eq. 1, based on average salinities and concentrations of nitrate + nitrite in AW found in the shelf stations V10, V12 and V14, in the top 100 m, and comparable values in IW, SW or AW, found in the Fjord stations Kb1-Kb5, and using 2.0 µmol kg^−1^ for the freshwater endmember.

Nitrate + nitrite Sources-Sinks (µmol kg^−1^)			
Year	AWs versus IW	AWs versus SW	AWs versus AWf
2011	**−2.0**	−1.2	–
2012	−4.9	**−5.0**	−5.2
2013	−3.7	**−4.2**	−2.6
2014	**−4.7**	**−4.0**	−2.6
2015	**−7.5**	**−7.7**	−4.4
2016	**−5.3**	**−5.0**	−2.5
2017	−3.3	**−3.2**	0.5
2018	−1.7	−1.8	1.9
2019	−4.0	**−3.7**	−2.9
2020	**−4.7**	**−5.2**	−1.8

See Methodology - **Freshwater inputs**, for nutrient concentrations in freshwater and Table S2 for water-mass classification. Subscripts s and f are used to distinguish AW in the shelf and in the fjord, respectively. No data is indicated by the dash “-” symbol. Values in bold correspond to significant concentration differences between the water masses (two-tailed *t*-test, *p* < 0.05).

**Table 2:** Biogeochemical DIC *Sources-Sinks* estimated with Eq. 1, based on average salinities and DIC concentrations in the AW found in the shelf stations V10, V12 and V14, in the top 100 m, and comparable values in IW, SW or AW found in the Fjord stations Kb1-Kb5.

		DIC Sources-Sinks (µmol kg^−1^)		
Year	Freshwater endmembers (µmol kg^−1^)	AWs versus IW	AWs versus SW	AWs versus AWf
2012	668	**−23.9**	−23.6	−17.4
2013	769	**−13.1**	−4.6	26.0
2014	615	**−34.5**	**−28.3**	−10.9
2015	1105	**−68.6**	**−72.8**	−5.1
2017	1180	**−37.4**	**−41.9**	4.7
2018	949	**−14.4**	**−23.9**	28.9
2019	834	**−27.9**	**−38.9**	−9.3
2020	555	**−10.4**	**−12.8**	14.6

DIC in the freshwater sources (endmembers) is the Y-intercept of a linear regression between salinity and DIC in SW (see text and Table S2 for water-mass classification). Subscripts s and f are used to distinguish AW in the shelf and in the fjord, respectively. Values in bold correspond to significant concentration differences between the water masses (two-tailed *t*-test, *p* < 0.05).

## Net ecosystem metabolism

**Fig. 5:**
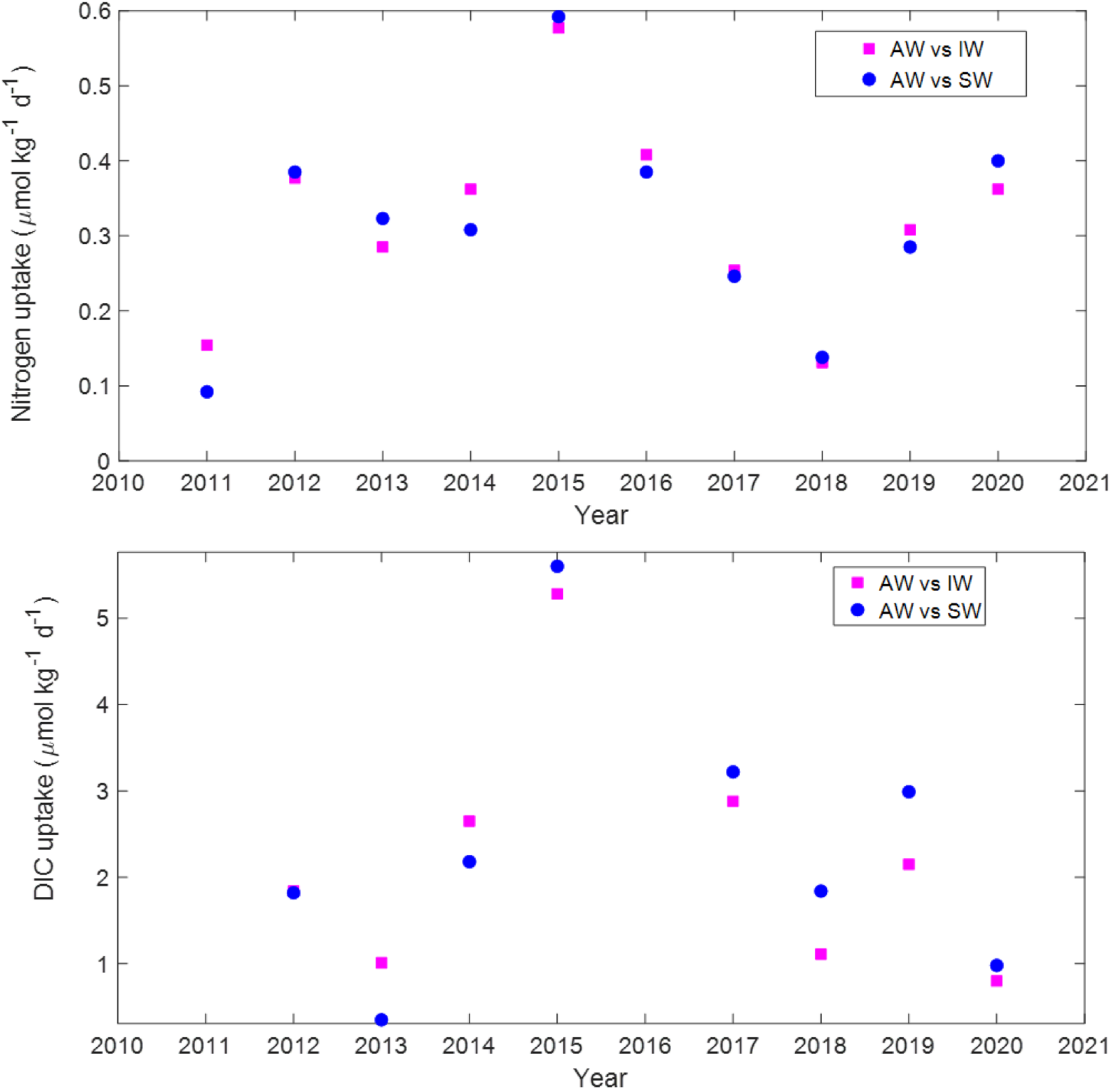
(**a**) and (**b**) Nitrate + nitrite and dissolved inorganic carbon (DIC) molar uptake rates, respectively (see text). We used a flushing time of 13 days which is the mean of the range obtained from our simulations (see Figs. S17a, S18a and S19a). Positive values indicate net drawdown inside the fjord (autotrophic fjord).

The average fjord flushing time was estimated to be ~ 13 days based on various model simulations (e.g. Figs. S17, S18, and S19). This value was used with data from Tables 1 and 2 to calculate molar drawdown rates for nitrate + nitrite, between 2011 and 2020, and DIC, between 2012 and 2015 and 2017–2020, due to data absence or limitations for 2011 and 2016 (see Methodology, **Net ecosystem metabolism** and Eq. 5). Here we focus on the contrasts between IW and SW in the fjord and AW on the shelf only. Nitrate + nitrite drawdown rates ranged from ~0.1 to 0.6 µmol N kg^−1^ d^−1^, whereas those for DIC ranged from ~ 0.3 to 5.6 µmol C kg^−1^ d^−1^. A strong correlation was observed between the nitrate + nitrite and DIC drawdown rates, except for the outliers in 2020 (Fig. 5a, b). The mean C: N uptake ratio was 7.2, which is close to the Redfield molar ratio of 6.6^25^. Vertically-integrated nitrate + nitrite and DIC drawdown rates for the upper 100 m were used as a proxy for NEM (refer to Methodology, **Net ecosystem metabolism**) and ranged from ~0.01 to 0.06 mol N m^−2^ d^−1^ and ~ 0.04 to 0.6 mol C m^−2^ d^−1^ (~ 0.1 to 0.8 g N m^−2^ d^−1^ and from ~ 0.4 to 6.7 g C m^−2^ d^−1^).

## Discussion

Our nitrogen budget is based on data collected between 2011 and 2023. It synthesizes all fluxes that we were able to quantify based on available data. It is not a complete budget, missing inputs/outputs associated with e.g. submarine groundwater discharges (SGD, e.g. Ref^26^), melting glacier ice [for example^27^], detritus particles, and the presence and migrations of biological groups such as fish and mammals. Whereas melting icebergs contain nutrient concentrations much lower than those found in river waters^27^SGD could be an important part of the terrestrial flux^26^. Moreover, while we computed nitrogen removal by birds, we were not able to quantify nitrogen returned to the fjord in the form of feces.

The ranges estimated for some of the fluxes are rather large, due to interannual variability. Furthermore, the uncertainty associated with the various fluxes and parameters used in our calculations may bias our averages. The dominant role of fjord-shelf exchanges aligns with findings from Greenland fjords with tidewater glaciers [for example^28^]. However, the differences between inputs and outputs from/to the sea are poorly constrained and play a determining role in defining the heterotrophic/autotrophic character of coastal ecosystems compared to riverine and atmospheric inputs [for example^1^].

Freshwater nutrient sources in Kongsfjorden include meltwater from surface and subglacial runoff and groundwater^29–31^with glacial meltwater being the most important source^32^. Snow melt dominates during the early melt season, creating higher nutrient concentrations due to the elution of atmospheric nitrate and ammonium from the snowpack^33^. As the season progresses, glacial ice melt dominates, supplemented by supra-permafrost groundwaters in non-glaciated areas of the watershed from mid-July onwards^31^. Submarine groundwater discharges and glacial runoff likely occur throughout the year [for example^34^ and references therein], but their magnitude is uncertain. The lack of measurements of freshwater fluxes and limited data about the chemical composition of these sources make it challenging to assess their contribution in forming fjord water masses such as SW and IW or to the fjord nutrient pools. Currently, this assessment is based on modeling^35^. However, freshwater dilution of seawater in Kongsfjorden is generally limited to a few parts per thousand, likely impacting water-column stratification and generating upwelling at the glacier fronts^36,37^ more than affecting fjord macronutrient concentrations directly (see below).

In summer, Kongsfjorden is dominated by AW and water masses resulting from its dilution with freshwater (IW and SW), with an increasing fraction of AW over the last ~ two decades, leading to an increase in fjord heat content and temperature^38^. The ratio of salinity values in the fjord to that of AW on the shelf (*S*: *S*_*AW*_) is a measure of the fractional reduction in salinity as shelf AW is diluted with glacial melt water. All these ratios are > 92%. Therefore, if we assume zero nitrate + nitrite concentrations in the glacier melt water and no biogeochemical sinks, we should obtain comparable fractional reductions for nitrate + nitrite or any other nutrients. However, most of the nutrient concentration ratios between SW, IW and fjord AW (AWf), compared with shelf AW (AWs), are < 77%, with most values being < 50%. Therefore, the fractional nitrate + nitrite reductions are much larger than the fractional salinity reductions. Considering that river endmembers have nitrate concentrations ranging from 0.4 to 8.91 µM^29,30,33,39–43^the effects of dilution should have been lower or even positive, emphasizing the importance of biogeochemical sink processes.

The above conclusion depends on the validity of the assumption that AW is diluted by mixing with meltwater when moving from the shelf into the fjord and not mixing with other water types, such as Arctic water masses (ArW). Otherwise, it would not be a valid endmember in our calculations. We argue that this assumption is valid because, in the data analyzed, we did not find any other water mass over the shelf or within Kongsfjorden except for TAW, lying usually below AW. Therefore, it would be rather unlikely that TAW could be the endmember to produce IW or SW. Moreover, nutrient concentration gradients in TAW are consistent with those found in AW, with decreasing concentrations towards the fjord, suggesting biogeochemical consumption.

The nutrient and DIC drawdowns were calculated based on snapshot observations inside and outside Kongsfjorden. While these estimates may be influenced by temporal differences in properties of AW, our analysis suggests that such biases are limited. Model simulations show that flushing time (FT*)* in most of Kongsfjorden ranges from 2 to ~ 15 days, considering the 60% reduction threshold (refer to Methodology - **Model description**, Text S5, and Figs. S17, S18 and S19). Conversely, nitrate + nitrite changes outside and inside Kongsfjorden (Fig. S16) are mainly seasonal, with minimal changes in periods comparable to FT. Moreover, a significantly positive correlation (0.71, p < < 0.001) was found between the time series represented in Fig. S16 for nitrate + nitrite values outside and within the fjord (stations F-S-4 and Ferry Box, respectively, refer also to Fig. 1). This shows that changes in Fram Strait correspond to changes in the fjord. These arguments will not rule out that our estimates are not biased but provide indication that temporal differences may not be determinant. Ideally, the data of marine endmember used in Eq. 1 should not only be spatially averaged, as done here using results from the shelf stations (V10, V12 and V14), but also temporarily averaged for a period comparable to the FT. This would consider nutrient variability in the marine endmember relevant for the properties of the water inside the fjord. However, we could not do the temporal averaging due to the lack of data. Future studies should consider lateral variability in nutrient/DIC concentrations as FT differs across the fjord (see Figs. 1 and S17–S19). However, the transect represents the middle of the fjord with smaller FT, where there is less time for the effects of biogeochemical processes to build up, reinforcing our conclusion about the nutrient/DIC sink role of the fjord.

Our results consistently show that fjord water masses (SW and IW) are a sink for nutrients/DIC relative to AW, except for ammonium where the fjord acts as a source. This pattern is consistent over different depth ranges (50 and 150 m). When 50 m is used, nutrient drawdown was lower due to surface-depleted water masses inside and outside the fjord and neglecting negative changes in nutrient concentrations from the shelf to the fjord occurring at greater depth. When we use 150 m, results are identical to our “standard” 100 m, because concentrations change less between 100 and 150 m depth, and there are several locations where calculations are limited to 100 m due to shallow bathymetry. Some of the negative changes in nutrient concentrations and the calculated *Source-Sinks* are based on water masses that do not differ significantly in nutrient composition. This does not mean the difference is not meaningful, but we lack statistical support. However, in most cases, AW differed significantly from IW and SW for nitrate + nitrite, DIC and silicic acid.

When comparing AW inside the fjord and on the shelf, trends were weaker or, in some cases, reversed. This probably reflects light limitation of nutrient uptake in AW below layers of SW and IW within the fjord. Moreover, mineralization and nitrification at depth could partly compensate for nutrient uptake.

Positive changes in ammonium are likely linked to input from subglacial discharge^43^ but are by far outweighed by negative changes in nitrate + nitrite. The presence of a biogeochemical sink for nutrients implies a positive, autotrophic summer NEM. Dissolved inorganic carbon concentration depends not only on dilution and biogeochemical sink/source processes but also on exchanges with the atmosphere. However, summer surface waters in Kongsfjorden are undersaturated, with a CO_2_ partial pressure (*p*CO_2_) < 400 µatm^23,44,45^indicating a net CO_2_ flux from the atmosphere (*p*CO_2_ > 400 µatm). This unaccounted negative flux might bias our DIC sink estimates, reinforcing our conclusion about autotrophic metabolism.

The biogeochemical sink is likely a result of phytoplankton and bacterial nutrient uptake [for example^27^] with an important contribution of the seaweed standing stock. This stock was estimated at 5000–18,000 tonnes DW, corresponding to 8–29 g C m^−2^ and comparable to peak pelagic protist standing stocks of 9 g C m^−2^ in June 2019 and 11.5 g C m^−2^ in May 2020^46^, emphasizing the importance of considering the benthic compartment in fjord production and metabolism, as recently also done in the Arctic Ocean^47^.

The magnitude of the biogeochemical sink may result from the retention of the ocean water inside the fjord, allowing the effect of biogeochemical processes to accumulate over time. Our “crude” NEM estimates (0.4 to 6.7 g C m^−2^ d^−1^) align with ranges observed in other fjord systems, e.g. in Greenland and Patagonia. Sejr et al.^48^ ] reported values ranging ~−5 to + 20 µmol O_2_ L^−1^ d^−1^ in a sub-Arctic Greenland fjord, based on incubation measurements. They defined these results as Net Community Production which we assume here to be comparable to NEM. If we consider a 1.3:1 carbon: oxygen stoichiometry, the upper end of their estimates (20 µmol O_2_ L^−1^ d^−1^ => ~26 µmol C L^−1^ d^−1^) is ~ 3× higher than the upper end of our estimates (~ 5.6 µmol C kg^−1^ d^−1^, see Fig. 5b). Using temporal differences in DIC concentration within given water masses, Crosswell et al.^49^ obtained values of Net Ecosystem Production between ~−15 and + 6 µmol C kg^−1^ d^−1^ along three Patagonian fjords, which is similar to our NEM estimates on the positive end of the range. In situ rates of carbon and nitrogen uptake by phytoplankton measured in Kongsfjorden in May 2017 were 0.3 to 1.1 g C m^−2^ d^−1^ and 0.13 to 0.17 g N m^−2^ d^−1^^50^, consistent with the lower range of our depth-integrated NEM estimates. On the West Spitsbergen shelf outside Kongsfjorden, net community production was estimated to be 0.11–0.16 mol C m^−2^ d^−1^, (1.3–1.9 g C m^−2^ d^−1^)^51^.

Limitation in seasonal data, especially outside the fjord, challenges *Sources-Sinks* calculations. The results of Calleja et al.^52^ for 2012 obtained only within the fjord (see Fig. 1c) show higher nutrient concentrations in the outer fjord during the spring bloom in May. Similar results were obtained for August and October but only with nitrate and phosphate below the maximum chlorophyll *a* fluorescence depth, which was shallower than 50 m and very likely located within the layers of SW/IW. The data shown in Fig. S16 from^18^ provide some evidence for a decrease in nitrate + nitrite concentrations towards the fjord in winter, spring and summer 2017. Therefore, available evidence suggests that Kongsfjorden may also be a nutrient sink in spring.

The high-frequency 5-year carbonate chemistry dataset by Gattuso et al.^45^ shows year-round CO_2_undersaturation in the surface. However, undersaturation may result from physical processes (e.g. surface water cooling) rather than carbon drawdown through autotrophic processes, as expected during the Arctic winter [for example^53^].

Our approach to separate dilution effects from biogeochemical processes provides a procedure to establish the sink-source role of a fjord which should be applicable to monitoring programs of other “estuarine-like” systems. This requires characterization of marine and freshwater endmembers sampled outside the system to avoid freshwater dilution effects. The details needed to characterize the freshwater endmembers depend on their relative importance in the nutrient budget.

The FT*-*based procedure to normalize biogeochemical *Sources-Sinks* to a relevant time scale is a crude, yet consistent way to normalize biogeochemical *Sources-Sinks*. The rationale is that if the time scale of *Sources-sinks* is larger than that of FT, their effect would hardly be noticed, since fjord waters would be flushed before their concentrations could be significantly changed by biogeochemical processes. If changes in *Sources-Sinks* take place over time concurrently with changes in FT, taking both into account helps disentangle the causes for the changes in the former.

The flushing time may be estimated with different methods such as the LOICZ method [i.e. equation 13 in Ref^54^] based on freshwater flows, the salinities of the inflowing seawater and the outflowing fjord water, and fjord volume. However, we also acknowledge that the main added value here is having a reproducible time scale.

Ongoing changes in the Arctic, such as increased temperatures, reduced sea ice, glacier retreat, and increased freshwater discharge are expected to influence NEM by altering nutrient distribution, light availability and, thereby, primary production, hence affecting important ecosystem services such as food provisioning and carbon storage [for examples^34,55^]. Such ecosystem-scale responses to anthropogenic perturbations justify ecosystem metabolism studies^11^. However, properly addressing the sign and magnitude of such changes depends on research focusing on the fundamental ecosystem processes of production and consumption and better characterization of the different nutrient sources.

Our results focus on the summer period, during which data availability is highest. In line with other fjord studies [for examples^47,56^ and considering the long dark seasons at the latitude of Kongsfjorden (79° N), we expect strong seasonal variability in nutrient sink-source processes and NEM, with heterotrophy dominating in winter and autumn and autotrophy dominating in spring and summer. Seasonal sampling surveys of the marine and the freshwater endmembers are necessary to address these issues. We do not attempt to spatially-resolve the sink-source processes and the role of autotrophy-heterotrophy in Kongsfjorden, but we expect it to be modulated largely by light limitation of primary production, controlled by the increasing turbidity towards the glacier fronts [e.g. Ref^43^].

Summing up all nitrogen fluxes summarized in Fig. 2a, except those between the fjord and the sea, indicates a loss of 1.2 tonnes d ^−1^. This value is relatively small compared to nitrogen NEM estimates, upscaled to the fjord area (~ 231 km^2^), which are in the range of 30–192 tonnes N d^−1^, suggesting that the metabolism of Kongsfjorden is mainly supported by exchange with the sea. This contrasts with results of Santos-Garcia et al.^57^who assigned 44% of the fjord’s nitrogen stock to terrestrial origin. However, smaller fluxes with significant temporal trends, such as increasing atmospheric nitrogen loads (Fig. S20), increased precipitation over Ny-Ålesund [for example^58^ and glacial run-off (Fig. S21), could have long-term impacts on the future nitrogen budget. Increases in glacier meltwater discharges may shift ecosystem metabolism towards heterotrophy due to its negative impact on light availability^59^while increases in upwelling at marine-terminating glacier fronts may enhance the nutrient supply to the surface, positively affecting the autotrophic processes some distance away from the turbid freshwater plumes [for example^43^.

### Methodology

For the purposes of the present study, we define Kongsfjorden as the fjord area east-southwest of the white line depicted in Fig. 1c, excluding Krossfjorden and the adjacent coastal area. The study site and its ocean context are briefly described below. Available data from Arctic marine ecosystems are biased towards the summer months due to the logistic constraints imposed by the cold and dark winter. This is also the case for Kongsfjorden (Fig. 1), where systematic physical and biogeochemical sampling has been mostly conducted during summer (e.g., Monitoring of Svalbard and Jan Mayen program [MOSJ; Ref  ^22^]). Here we also analyze data collected in other seasons, but at a lower spatial resolution than the summer data. Thus, our focus is on the summer season (July and August). In this study, we use the term “nutrients/DIC” to include inorganic forms of nitrogen (ammonium, nitrate, and nitrite), phosphate, silicic acid, and inorganic carbon (dissolved inorganic carbon, DIC). By combining fluxes computed from available data and/or based on model results, we quantified the contribution of different processes to the fjord nitrogen budget. The fluxes considered are water exchanges with the open ocean, freshwater inputs, atmospheric inputs, and biological inputs/outputs (e.g., advection of planktonic organisms, and bird feeding), as detailed below (see—Methodology,** Nitrogen budget**). The nitrogen budget mentioned above is poorly constrained regarding the net effect of some of the major fluxes, making it challenging to evaluate the fjord’s nutrient/DIC sink/source role (cf.—**Discussion**). An alternative way to gain insight into this role is to analyze horizontal gradients in nutrient/DIC concentrations—this requires data from within and beyond the fjord system. The main challenge for analyzing horizontal gradients is to distinguish changes in nutrient/DIC due to the mixing of different water masses (i.e., purely physical processes) from biogeochemical changes such as nutrient and carbon uptake and release by organisms. We use a methodology to quantify the biogeochemical sink/sources when water masses entering the fjord are modified by mixing with freshwater (see—Methodology,** Analysis of nutrients/DIC concentration gradients and quantification of biogeochemical sinks/sources**). However, the available data do not allow us to compute the time scale of biogeochemical sinks/sources, as we can only calculate the amount of nutrient/DIC added to or removed from a water mass. Without a time reference, it is not possible to quantify NEM. Therefore, we use the flushing time (FT) (e.g. Ref.^60^) as a proxy for the mentioned timescale. We estimated FT using the three-dimensional hydrodynamic model K160_bgc (see—Methodology—**Net ecosystem metabolism, Model description** and Text S5). When possible, we express concentration in μmol kg^-1^. However, in some cases, when referring to published data, we follow the original units in μmol L^-1^ (μM). 

## Study site

Kongsfjorden is in the Svalbard archipelago (West Spitsbergen, ~ 79^o^N 11–13^o^E) (Fig. 1). The fjord is largely influenced by the cold and fresh Arctic Water (ArW) carried by the Spitsbergen Polar Current (SPC) on the West Spitsbergen Shelf (WSS), and the warm and salty Atlantic Water (AW) transported by the West Spitsbergen Current (WSC) along the shelf slope^61^ (Fig. 1a). Six tidewater glaciers supply sub-glacial freshwater inflows at depth into the fjord, while a multitude of relatively small rivers and streams supply freshwater from several glaciated and non-glaciated basins into the surface of the fjord (Fig. 1c)^35,62^. Many studies on the hydrography and biogeochemistry of Kongsfjorden are available [for example^15^ and references therein]. Some of these studies emphasize the increasing presence of AW, explaining part of the positive trend in summer water temperatures in the last ~ 2 decades [for example^38^], and its influence on the fjord’s biogeochemistry^23^ and the planktonic ecosystem^46^. Intrusions of AW in Kongsfjorden are more frequent in summer, but also occur in winter^23,63^. The decrease in winter/spring sea-ice extent in recent decades is one of the most remarkable changes in this fjord^64^. Seasonal sampling surveys conducted in Kongsfjorden^46,65^ showed the occurrence of a phytoplankton spring bloom between late April and early June that ceased when silicic acid and nitrate reached limiting concentrations. Strong stratification developed in summer due to freshwater runoff and surface-water heating. Summer phytoplankton blooms were observed on some occasions, apparently linked with elevated ammonium [for example^43^]. There was a rapid decline of chlorophyll *a* in the autumn, which continued during the polar night^65^.

The benthic compartment of Kongsfjorden includes barren rock, kelp beds, gravel and soft bottom, which is the most common substrate type^66^. Shallow benthic communities show a high level of omnivory and seem resilient to seasonal changes^67^.

## Nitrogen budget

We calculated nitrogen inputs and outputs from various datasets (Table S1) and literature sources specified below. When combining results from different data sources, we simply averaged them and evaluated their total range.

### Atmospheric deposition

Nitrate and ammonium-nitrogen deposition to the fjord surface were calculated from precipitation data (mm) (https://seklima.met.no/observations*)* and dissolved and aerosol (particulate) nitrogen concentrations (https://ebas-data.nilu.no/Default.aspx*)* for the period 2011–2020, from Ny-Ålesund station, in Svalbard, Norway. The concentration data are usually based on periods of ~ 7 days. Therefore, for each period, the total precipitation was integrated and then multiplied by the concentration data to obtain nitrogen deposition in its various forms (nitrate dissolved and particulate and dissolved ammonium). Then, an average daily deposition of nitrogen was obtained. This value was then used to upscale deposition for the fjord area (231.5 km^2^), delimited by the white line in Fig. 1c. Results from nitrogen aerosol and ammonium precipitation were orders of magnitude lower than those for nitrate precipitation, and were thus not included in the budget calculations.

### Freshwater inputs

Nutrient fluxes associated with riverine transport were estimated from the product of total runoff volume and the nutrient concentrations reported by studies of glacial rivers in Kongsfjorden basins, with a near-complete coverage of the peak runoff period (July and August), from 1991 to 2010 [Refs^29,30,33,39–43^]. These yielded the following concentrations (means ± standard deviations) for ammonium, nitrate, phosphate, and silicic acid, respectively: 1.44 ± 1.69, 2.00 ± 1.24, 0.064 ± 0.056, and 9.77 ± 6.24 µM. These results were based on 759, 448, 65, and 741 samples, respectively. DIC in the freshwater sources (endmembers) was calculated from the Y-intercept of linear regressions between salinity and DIC in SW. River runoff was obtained from the model described in^35^ and is based on the glacier energy balance and the routing of the water along the various glacier basins. Therefore, this estimate of freshwater inputs considers only surface and subglacial outflows and neglects other freshwater sources such as ground-ice thaw from permafrost, and ice melting from landfast sea ice and icebergs calved from marine-terminating glaciers. In the case of the latter, we expect low nutrient loads considering the data presented by Cantoni et al.^44^.

### Inputs/outputs associated with fjord-ocean physical exchanges of dissolved nitrogen

Fjord-ocean inputs/outputs were estimated by (i) time-averaging the volume transport across the transect depicted in Fig. 1c (white line delimiting Kongsfjorden) from model simulations (see - Methodology - **Model description** and Text S5); (ii) multiplying the volume transport by the average inorganic nitrogen concentrations based on measurements close to the same transect and at various depths. For this purpose, we used data from Calleja et al.^52^ from surveys conducted in Kongsfjorden in 2012 and MOSJ data (see Table S1). We selected vertically weight-averaged ammonium, nitrate, and nitrite values for the “outer fjord” (see Fig. 1; Table 1 in the Supplementary material of Calleja et al.^52^). We combined these results with similar depth averages obtained from the MOSJ data for stations Kb0 and Kb1. We summed the weighted averages to obtain a total inorganic nitrogen concentration, which was then multiplied by the volume transport. We have chosen not to use the other datasets listed in Table S1 for this specific purpose because they were obtained for areas distant from the white line depicted in Fig. 1c (the interface between the fjord and sea).

### Biological stocks and inputs/outputs

We estimated the contribution of nitrification and denitrification in the water column to the nitrate pool based on field and incubation experiments from samples collected between stations Kb1 to Kb5 (Fig. 1). Details are provided in Supporting information Text S1 and Tables S3 and S4. Denitrification fluxes from the sediments were based on^21^.

We computed phytoplankton and zooplankton nitrogen fluxes based on stock values for these organisms in the outer region of Kongsfjorden and the volume transport across the transect depicted in Fig. 1c (white line delimiting Kongsfjorden) from model simulations (see - Methodology - **Model description**, Text S2 and Text S5). Kelp standing stock was estimated using seafloor light and biomass data as detailed in Text S3. We estimated the nitrogen consumption by birds as detailed in Text S4.

### Hydrography, nutrients, and dissolved inorganic carbon in kongsfjorden, the shelf and the Eastern Fram Strait

We used published datasets^18,52,65,68–71^ (detailed in Table S1) to describe the spatial patterns of water masses and nutrients/DIC between the shelf and the inner parts of Kongsfjorden. Despite our focus on Kongsfjorden, we also report data obtained on the shelf to provide the physico-chemical context of water masses found in Kongsfjorden (Figs. 1c, d). The MOSJ data sets [for examples^22,68–72^]. include vertical profiles taken systematically along the same transect and at the same time of the year (end of July, beginning of August) for a total of 10 years. The other datasets were obtained in different seasons with a good overlap with some of the stations sampled during the MOSJ transects. However, these included mostly stations within the fjord. These datasets were supplemented with data available in the literature, as specified in the text.

### Analysis of nutrients/DIC concentration gradients and quantification of biogeochemical sinks/sources

The MOSJ data specified in Table S1 were used to produce transect plots from inner Kongsfjorden, and across the shelf for the summer season. These plots (Figs. 3 and 4 and S7, S10, S12, S14) contain information about the spatial distribution of different water masses classified with the envelopes shown in Table S2, and about nutrient/DIC concentrations, including their vertical and horizontal variability. They were obtained after interpolating data collected along CTD (conductivity, temperature, depth) casts in the stations shown in Figs. 1b, c. We plotted results for the top 150 m since our focus was on the surface waters. From these plots, we identified the ocean endmember, i.e., the water mass on the ocean side that is the main origin of the water masses in Kongsfjorden during summer (i.e., AW from the WSC). Atlantic Water may become Transformed Atlantic Water (TAW) when mixed with ArW from the SPC. The AW or TAW mixes with Surface Water (SW) producing Intermediate Water (IW) [Ref^24^ and Table S2].

If we assume that SW and IW result solely from the dilution of AW with freshwater and if we know the salinity and nutrient concentration in AW and in the freshwater, we may estimate the expected nutrient concentration in SW and IW from their salinities, after fitting a regression line to a mixing diagram [for examples^73–75^]. The difference between the observed concentration and that predicted from the mixing diagram reflects biogeochemical transformations, as shown in Eq. 1 (see Fig. S1 for details).

1 $$\:Sources-Sinks=\varDelta\:C={C}_{obs}-{C}_{cons}={C}_{obs}-\left[{C}_{0}+\left({S}_{obs}-{S}_{0}\right).\left(\frac{{C}_{AW}-{C}_{0}}{{S}_{AW}-{S}_{0}}\right)\right]$$

Where *Sobs* and *Cobs* are the observed average salinity and nutrient concentration within the fjord (in SW, IW, or AW), *Ccons* is the expected concentration in the absence of sources and sinks (calculated with the term in square brackets - a linear regression between concentration and salinity), *S*_*AW*_ and *C*_*AW*_ are the average salinity and nutrient concentrations in AW on the shelf, and *S*_*0*_ and *C*_*0*_ are the average salinity and nutrient concentrations in freshwater. We also distinguish between AW on the shelf and within the fjord (referred to as AWs and AWf, respectively) because the latter may be diluted in relation to the former without being transformed into SW or IW. In the case of a conservative tracer (concentration changes only because of dilution), *Sources-Sinks* = 0. In the case of a non-conservative tracer, such as nutrients and DIC, *Sources-Sinks* is the net change resulting from biogeochemical production and consumption processes. If the tracer is or contains a gas, the last term of Eq. 1 should include the net exchanges with the atmosphere. We used nutrient and DIC concentrations in the fjord and on the shelf from the MOSJ dataset (Table S1) and river concentrations from data described above in Nitrogen budget - **Freshwater inputs**.

When using Eq. 1, the depth range used to obtain the nutrient/DIC concentrations affects the calculations. If a large depth range is considered, it is likely that any surface differences between the water masses will be diluted by the relative homogeneity of concentrations in deep waters. Therefore, we obtained vertically-averaged concentrations for the top 100 m where more variability was observed in nutrient/DIC concentrations. Moreover, the depth of inner Kongsfjorden reaches ~ 100 m or less and, if we used a larger depth range, average concentrations along the fjord would not be comparable, considering the large depth range along the sampled transect (Figs. 1b, c). In our calculations, we assumed that salinity in glacier melt water is zero.

We tested the significance of the differences between the arithmetic means of the water masses used for the calculations of the sources-sinks (AWs versus AWf, AWs versus IW and AWs versus SW) using two-tailed *t*-tests. If the concentration of a given nutrient does not differ significantly between two water masses, we cannot statistically support any difference between them. This is not the ideal solution since the variance in each water mass was estimated from samples collected at different places and depths and hence the samples do not represent true replicates for statistical testing.

### Net ecosystem metabolism

There is no timescale associated with Eq. 1. Therefore, this equation provides an estimate of the average net effect of sources and sinks, and the relative role of heterotrophy/autotrophy, without estimating a rate and thereafter NEM.

Equation (2) provides a simplistic description of the concentration changes within the fjord for a dissolved tracer as a function of time:

2 $$\:\frac{\varDelta\:C}{\varDelta\:t}=\frac{Q\left({C}_{sea}-{C}_{fjord}\right)}{V}+\frac{{Q}_{0}\left({C}_{river}-{C}_{fjord}\right)}{V}+\frac{Sources-Sinks}{\varDelta\:t}$$

Where *Q* represents the flow exchanges with the shelf (m^3^s^−1^), *V* (m^3^) is the fjord volume (in our case, this is limited to depths ≤ 100 m, see previous section), *Q*_*0*_ is the total river and glacial freshwater flux, and *C*_*sea*_, *C*_*fjord *_*and C*_*river*_ (mg m^−3^ or mmol m^−3^) represent the tracer concentration in the shelf, the fjord, and the freshwater, respectively.

We will show that exchanges with the sea are many orders of magnitude larger than river flows. Therefore, we may neglect the second term on the right of Eq. 2. Assuming steady state, we can simplify Eq. 2 to:

3 $$\:\frac{Q\left({C}_{sea}-{C}_{fjord}\right)}{V}+\frac{Sources-Sinks}{\varDelta\:t}\approx\:0$$

Which may be rewritten as:

4 $$\:\frac{Sources-Sinks}{\varDelta\:t}\approx\:-\frac{{C}_{sea}-{C}_{fjord}}{FT}$$

This implies that biogeochemical sinks/sources must match exchanges with the sea for the steady-state condition to hold. It also implies that biogeochemical sinks/sources may be estimated by dividing observed concentration differences between the fjord and the sea by the fjord flushing time (*V/Q =*FT; for example^60^). Therefore, Eq. 5 is used to calculate NEM:

5 $$\:NEM\approx\:-\frac{Sources-Sinks}{FT}$$

The steady-state assumption as well as the “whole fjord” treatment in Eqs. 2–5 are grand simplifications of the real world considering the temporal and the spatial variability of water column properties. Our approach is simply intended to apply a meaningful and reproducible timescale to relate to spatial nutrient/DIC changes. The FT within the area bounded by the white line shown in Fig. 1c was estimated from an ocean model (Text S5).

### Model description

The Regional Ocean Modeling System (ROMS) (https://www.myroms.org/*) *is used for modeling Kongsfjorden and its adjacent coastal area (Fig. S2) and to calculate FT – for simplicity, the time required to flush out 60% of the original amount of a tracer (refer Text S5 and references therein for a detailed definition of FT and its calculation from model simulations). The present model (K160_bgc) is based on a one-way nested model system like that described in Sundfjord et al.^76^ with differences specified in Texts S5 and S6.

## Electronic supplementary material

Below is the link to the electronic supplementary material.

Supplementary Material 1

## Data Availability

The datasets generated and/or analysed during the current study are available in the Norwegian Polar Data Centre, the Norwegian Centre for Climate Services, the EBAS database, and PANGEA repositories (https://data.npolar.no/dataset, https://seklima.met.no/observations, https://ebas-data.nilu.no/Default.aspx, https://www.pangaea.de/, respectively), at the following links; https://data.npolar.no/dataset/e53eae53-147a-45df-b473-917bb5ba1ed4, https://data.npolar.no/dataset/87ca4acd-69bc-4498-a8ea-db6fde729bb3, https://data.npolar.no/dataset/dc75c696-8a0a-4035-82e1-4e7f6cdfb05a, https://data.npolar.no/dataset/4d4de169-bf39-4245-b57e-1552c6e9f19f, https://doi.pangaea.de/10.1594/PANGAEA.936749, https://doi.pangaea.de/10.1594/PANGAEA.931854.
