# Supplementary material for: Ecosystem metabolism and nitrogen budget of a glacial Fjord in the Arctic
Source: Sci Rep. 2025 Jul 2;15:22946. doi: 10.1038/s41598-025-06953-3 (PMC12215447; doi:10.1038/s41598-025-06953-3)
Supplement: Supplementary file 1 — Supplementary Material 1 [file 41598_2025_6953_MOESM1_ESM.pdf]

Supporting Information for

Ecosystem metabolism and nitrogen budget of a glacial fjord in the Arctic

Pedro Duarte[1*], Laura Castro de la Guardia[2], Philipp Assmy[1], Anette Wold[1], Agneta Fransson[1], Melissa Chierici[3], Allison Bailey[1], Andrew Hodson[4,5], Andreas Alexander[6], Catarina Magalhães[7], Geir Wing Gabrielsen[1], Jon Albretsen[8], Lukas Frank[4,9], Sarat Chandra Tripathy[10], Carlos Smerdou[11], Francisco J.L. Gordillo[11], Pablo Cobos[11], David Velázquez[12], Peter Convey[13,14], Francesco De Rovere[15,16], Haakon Hop[1]

[1] Norwegian Polar Institute, Fram Centre, 9296 Tromsø, Norway

[2] Scottish Association for Marine Science (SAMS) Dunstaffnage Marine Laboratory, Oban PA37 1QA

[3] Institute of Marine Research, 9007 Tromsø, Norway

[4] University Centre in Svalbard, 9171 Longyearbyen, Norway

[5] Department of Civil Engineering and Environmental Sciences, Western Norway University of Applied Sciences, Sogndal Campus, 5020 Bergen, Norway

[6] Norwegian Water Resources and Energy Directorate

[7] Interdisciplinary Centre of Marine and Environmental Research, University of Porto, Terminal de Cruzeiros do Porto de Leixões, Av. General Norton de Matos s/n 4450-208 Porto, Portugal

[8] Institute of Marine Research, PO Box 1870, 5817 Bergen, Norway

[9] University of Bergen, PO Box 7800, 5020 Bergen, Norway

[10] National Centre for Polar and Ocean Research, Vasco-da-Gama, Goa-403804, India

[11] University of Málaga, Faculty of Sciences, 29071 Málaga, Spain

[12] Universidad Autonoma de Madrid, Dept. of Biology, 28049 Madrid, Spain

[13] British Antarctic Survey, NERC, Cambridge, UK

[14] Department of Zoology, University of Johannesburg, South Africa

[15] Ca' Foscari University of Venice, Department of Environmental Sciences, Informatics and Statistics, Venezia, Italy

[16] Consiglio Nazionale delle Ricerche, Istituto di Scienze Polari (CNR-ISP), Bologna, Italy

Contents of this file

Text S1 – Upscaling of denitrification and nitrification

Text S2 - Phyto- and zooplankton fluxes

Text S3 - Kelp distribution and standing stock

Text S4 – Nitrogen consumption by birds

Text S5 - K160_bgc model

Text S6 - S500 model

Tables S1-S9

Figures S1 to S21

References

Text S1

Upscaling of denitrification and nitrification

Summer and annual nitrification and denitrification rates were upscaled from spring field observations[1]. These authors performed field measurements of denitrification and nitrification on three days in March (beginning, mid, and end of March), in the upper 50 m of the water column. Water samples were collected from stations Kb1 to Kb5 (Fig. 1). The water temperature ranged from -2.8 to 0 °C. Nitrification (denitrification) rates were measured by chemically blocking denitrification (nitrification) during incubation at ambient temperature (2 - 0.5°C). Additional incubation experiments were run to determine the sensitivity of both processes to daylight. Denitrification rates were not sensitive to daylight length, but nitrification was inhibited with increasing daylight hours (Fig. 6 in Ref. [1] and Table S3). Therefore, we assume that nitrification at the top 50 m is active only during the polar night (criteria for polar night was 24h dark), which was estimated as 115 days / 2760 hours, using the online tool https://aa.usno.navy.mil/data/Dur_OneYear. Nitrification was assumed to be active throughout the year (8760 h) in the volume of water below 50 m, which is likely to receive no light [e.g., Refs. [2, 3]). Denitrification, on the other hand, was assumed to be active throughout the year at a constant rate below and above 50 m. These rates and the fjord volume, estimated using the K160_bgc model grid and bathymetry within the area depicted in Fig. 1c (Kongsfjorden as delimited by the white line), were used to upscale nitrification and denitrification spatially and temporarily, based on field and laboratory incubations (Table S4). We consider the rates to be constant throughout the year, although temperature likely affects these rates. As shown by Krishnan et al. [1], nitrification and denitrification rates are positively related to temperature and can double at 12°C water temperature.

$$TN = [Vol \times Rate \times H] \quad (S1)$$

where *TN* is the total nitrogen contribution from each process, *Vol* is the volume of the water layer of interest, *H* is the number of hours per year (or polar night in the case of nitrification above 50 m) or in summer (July-August).

Text S2

Phyto- and zooplankton fluxes

We estimated phytoplankton and zooplankton nitrogen fluxes based on stock values for these organisms in the outer region of Kongsfjorden (Table 1 in Ref. [4], and references therein) that were converted to concentrations based on known depths, and MOSJ chlorophyll concentration data for stations Kb0 and Kb1. These data were combined with the volume transport across the transect depicted in Fig. 1c (white line delimiting Kongsfjorden) from model simulations (see - Methodology - Model description and Text S5), similar to the computation of nutrient fluxes. Chlorophyll was converted to carbon using a C: Chlorophyll ratio of 50 [e.g. Ref. [6]]. Phytoplankton stocks ranged from 0.35 to 5.3 g C m^{-2} and were converted to nitrogen assuming the Redfield mass ratio (C: N=5.7). The AMUST-KB3 dataset (Table S1) includes chlorophyll concentrations, particulate organic carbon (POC), and nitrogen (PON) for several samples. The average C: Chlorophyll ratio was 147 and the average POC: PON was 7.8. However, it is difficult to assess the contribution of detritus and zooplankton to these ratios. Therefore, we have chosen to use the literature values mentioned above. Regarding zooplankton, we used a stock of 12 g dry weight (DW) m^{-2}, obtained from samples collected at station Kb0 (Table 6 in Ref. [4]). We also used more recent estimates based on sampling with a WP2 net (mesh size of 180 µm) at station Kb1 (Fig. 1c) in 2020, 2021, 2022, and 2023. The average zooplankton stock was

estimated as 19 g DW m^{-2}, using a dry mass conversion factor derived from literature values (see Supplementary Table 1 in Ref. [5]). Dry weight values were converted to nitrogen using 0.08 g N g $(DW)^{-1}$ [6].

Text S3

Kelp distribution and standing stock

Kelp standing stock was estimated using published high-resolution seafloor light[7] and summer biomass data along a depth transect (Tables S1 and S2 in Ref. [8]). We first estimated the area of potential kelp presence using the light threshold of 47 mol photons m^{-2} yr^{-1} [Ref. [9]]. Then, we used depth-to-biomass data to obtain a relationship, which was applied to the potential kelp areas to estimate the biomass gradient from the nearshore to greater depths (details in Table S5, and Fig. S6). Finally, biomass was integrated over depth and area to obtain the standing stock existing within the fjord area defined in Fig. 1c. The kelp bottom percentage coverage variability was used as a proxy for biomass variability to obtain the standing stock range. The carbon and the nitrogen content in the dry weight of kelp was estimated using published averaged values for *Saccharina latissima*: 36% C and 1.4% N of DW, which were derived from seven fjords in the Arctic (Table 1 in Ref. [10]).

The depth-biomass relationship was established using averaged dry weight measurements (g DW m^{-2}) of three kelp species (*Alaria esculenta*, *Saccharina latissima* and *Laminaria digitata*) in 2012 and 2013 at discrete depths (0, 2.5, 5, 10, and 15 m) along an inshore to offshore transect in Hansneset, Kongsfjorden (Ref. [8], Tables S1 and S2). The curve fitting method was the piecewise polynomial (function PCHIP in Matlab https://www.mathworks.com/help/matlab/ref/pchip.html; Refs. [11, 12]).

The curve is constructed by fitting biomass data to each depth range (piece) independently. Each piece thus represents a section of the curve, having its own set of coefficients (Table S5). In our example (Fig. S6) we added two artificial points at 20 and 50 m depth to force the curve to a 0 value. The data were then interpolated from 0 to 50 m depth at 0.5 m intervals using the obtained PCHIP function for each depth range.

The kelp bottom coverage in the region where the biomass data was obtained was estimated at 45% (Area n. 6 in Table 1 of Kruss et al. [13]) and it varied between 17 and 62% in other regions along the coast of Kongsfjorden (Areas n. 5 and n. 2, respectively, in Table 1 of Kruss et al. [13]). Therefore, the 17 to 62% range in kelp cover becomes 38% and 135% when scaled to the percentage cover at Hansneset. Thus, the standing stock was multiplied by 0.38 and 1.35 to obtain the lower and the upper range, respectively. In this way we adjust our potential estimates to reflect the scarcity of hard substrata and/or the presence of sea urchins (Kruss et al. [13]).

Text S4

Nitrogen consumption by birds

We used our own published data on daily energy expenditure for 11 seabird species in Kongsfjorden [e.g. Refs. [14, 15]]. The estimate was made for breeders, non-breeders, and chicks during their residence time in Kongsfjorden. The annual food requirement for the Kongsfjorden seabird community is approximately 426 tonnes y^{-1} (wet weight) (considering the most recent bird population estimates). Since ~70 % of the food is taken from Kongsfjorden, birds consume ~298 tonnes y^{-1} (wet weight) of food from the fjord. The carbon consumption was estimated from the food consumption, assuming a dry mass: wet mass ratio of 1/3 and a carbon content of 40 % [4]. Carbon consumption was converted to nitrogen assuming a C: N mass ratio of 3.4, based

on values for polar cod (*Boreogadus saida*) and capelin (*Mallotus villosus*)[16], which are common seabird prey in Kongsfjorden[17]. To obtain summer daily consumption rates, we assumed that most seabirds stay in the fjord from 15 May to 15 August[4].

Text S5

K160_bgc model

The basic properties of the 3D numerical model setup, including the extent of the model domain, horizontal resolution (160 m), number of vertical (sigma) layers (35), and bathymetry used in this study, are the same as described in the "present-day scenario" by Torsvik et al. [Ref. [18]] (Fig. S2a).

Our model configuration includes changes in relation to the that described by Sundfjord et al.[19], detailed by [18] (i)-(iii), plus those specified below (iv-v): (i) the model domain extends further west and not so far to the north as the configuration described in [19]; (ii) the coastline has been adjusted using the S100 Svalbard map dataset[20]; (iii) the terminuses of the tidewater glaciers correspond to their locations in 2009; (iv) an analytical plume model was coupled with the ocean model to simulate the upwelling of the subglacial discharge plumes and the associated entrainment of fjord water[21] and (v) we use spatially variable Jerlov optical water types[22], based on photosynthetically active radiation data and light extinction coefficients reported in [23] (Fig. S2b). We used the same atmospheric and oceanic forcings, and boundary conditions described in [18].

The high-resolution atmospheric fields used to force the model over the period Sept. 2006 – Sept. 2007 were provided by the Weather Research and Forecasting model (WRF), developed by

the National Center of Atmospheric Research (NCAR). The WRF model is a state-of-the-art numerical weather prediction model[24], and its implementation includes a domain with a horizontal grid resolution of 3 km. The model was initialized with an analysis (on a 0.25° × 0.25° grid) of upper air and surface data from the European Centre for Medium-Range Weather Forecasts (ECMWF), and lateral boundary values were updated every 6th hour during integration.

Ocean boundary conditions were obtained from a two-layer one-way nested model system comprising a Pan-Arctic model at 4 km horizontal resolution (A4 model) and a smaller model at 800-m resolution (S800 model), described in [19, 25]. Daily averages from the A4 model are used to provide open boundary conditions for the S800 model domain, and tidal forcing for both A4 and S800 is provided by the global tidal model TPXO v7.2 [Ref. [26]; https://www.tpxo.net/global]. Hourly output from S800 is used to define boundary conditions for K160_bgc, hence the inner nested layer does not make use of a separate tidal model. The K160_bgc simulations are carried out with a 4-month spin-up period (Sept.–Dec. 2006), followed by a 9-month simulation period (Jan.–Sept. 2007). The reason for using these conditions for 2007 is the availability of many salinity-temperature profiles in Kongsfjorden for that year, which are adequate for evaluating simulated salinity-temperature profiles.

We plotted a sample of the vertical profiles of salinity and temperature bias for some of the stations depicted in Fig. 1. Generally, temperature and salinity biases were $< 1\ ^{o}C$ and < 0.2, respectively (Fig. S3). Taylor diagrams[27] were produced based on comparisons between model results and > 300 CTD salinity and temperature vertical profiles[28] (Fig. S4). We also made comparisons with the results of the S800 model providing the boundaries to the K160_bgc model. The Centred Root Mean Square Difference of the former is larger than that of the latter,

whereas the opposite is true for the correlation coefficient between observations and model results. Moreover, the standard deviation of the K160_bgc model is closer to that of the observations. The results show that the higher resolution K160_bgc model performs better than the S800 model and that part of its bias is "inherited" through the S800 boundary conditions.

The K160_bgc simulation evaluated here was also used to compute the fjord flushing time (FT), as described below. The FT is a bulk parameter describing the exchange characteristics of a waterbody without identifying the underlying physical processes, their relative importance, or their spatial variability (e.g. Ref. [29, 30]).There are different ways to calculate FT [29], such as *V/Q* (where V is the system volume and Q is the volumetric flow rate). However, this approach assumes that the waterbody is a continuously stirred tank reactor[29]. To avoid such simplified assumption, we used forward simulations to compute FT as described in [30] and detailed below.

To estimate FT, K160_bgc simulations were run for spring/summer periods (after a spin-up run of > 2 months) using a passive tracer with an initial concentration of 1 inside the fjord (area delimited by the white line in Fig. 1c) and above 100 m depth. Elsewhere, the concentration of this passive tracer was set at zero. This depth range was chosen for compatibility with the range used to calculate tracer concentration gradients and NEM (cf. - Methodology - Analysis of nutrients/DIC concentration gradients and quantification of biogeochemical sinks/sources). Then we ran the model and monitored the tracer decrease over time (e.g. Ref. [30]). The tracer concentration decreases with time, but it never reaches zero. Therefore, some threshold concentration must be chosen as a criterium to calculate FT. Here we set it at 40% of the original concentration and FT was calculated as the number of days required to flush out 60% of the original amount of the tracer, following Edwards and Sharples' study in a fjord in Scotland[31] (equations S1 and S2). We expect the FT to vary with tidal amplitudes, freshwater discharges[29]

and boundary conditions. Therefore, to consider this variability, we ran more simulations, using more recent years. For that purpose, we used boundaries provided by the S500 model (Text S6). The availability of boundary conditions from larger scale simulations limited our own simulations to spring-summer 2007 and 2019.

$$TracerContent_{day} = \sum_i \sum_j \sum_k \left(x_{ijk} V_{ijk} \right) \quad \text{(S1)}$$

Where *i*, *j*, and *k* are grid indexes used to sum up the tracer contents in each model grid cell for each simulated day.

$$TracerFraction_{day} = \frac{TracerContent_{day}}{TracerContent_1} \quad \text{(S2)}$$

$TracerContent_1$ is the amount of tracer at the beginning of the simulation.

Text S6

S500 model

The S500-model system (Fig. S5) is used to provide the open boundary conditions for the K160_bgc for 2019 simulations. The setup is based on ROMS-version 3.5 [Refs. [32, 33, 34]], including a simple sea-ice model introduced by Budgell[35]. The bathymetry-following vertical coordinate system is defined by the same 35 sigma-levels used in K160_bgc, while the horizontal model grid has a fixed resolution of 500 m and covers the entire Svalbard archipelago. Lateral boundary conditions and tidal forcing are applied using data from the Norwegian Meteorological Institute (MET Norway)'s operational ocean forecasting model Barents2.5[36]. Atmospheric forcing time series stem from MET Norway's operational atmospheric forecasting model AROME-Arctic AA, 2.5 km horizontal resolution, Ref. [37]. Daily runoffs from freshwater

sources are based on simulations with the CryoGrid land surface model, forced with AROME-Arctic as well [Refs. [38, 39]].

When using boundaries provided by the S500 model for the K160_bgc simulations we use the same forcings of the former model, except for the glacier discharges that were taken from [40].

Tables S1 to S9

Table S1. Datasets used in this study.

Data sets	Survey	Dates	References
Calleja et al.	Seasonal transect cruises (nutrient profiles along Kongsfjorden)	May, August, and October 2012	41
Monitoring of Svalbard and Jan Mayen program (MOSJ)	Transect cruises (CTD and nutrient and DIC profiles between the deep eastern Fram Strait and inner Kongsfjorden)	July-August 2011-2020	42, 43, 44, 45
Torres-Valdes et al.	Moorings with remote access nutrient samplers	August 2016 – August 2017	46
AMUST-KB3	Biogeochemical and ecophysiological monitoring at station Kb3 in Kongsfjorden	April and May 2014, 2016-2018	47
AMUST-transects	Transect cruises (CTD and nutrient profiles)	April and May 2016-2018 and 2021	
AWIPEV underwater observatory	Ferry box system measuring nutrient concentrations	April 2014-May 2018	48

Table S2. Definitions of water masses in Kongsfjorden following [49].

Water mass	Abbreviation	Potential temperature (ºC)	Salinity (psu)
Atlantic water	AW	>3.0	>34.65
Arctic water	ArW	-1.5 to 1.0	34.30 to 34.80
Winter-cooled water	WCW	<-0.5	34.40 to 35.00
Local water	LW	-0.5 to 1.0	34.30 to 34.85
Surface water	SW	>1.0	<34.00
Transformed Atlantic water	TAW	1.0 to 3.0	>34.65
Intermediate water	IW	>1.0	34.00 to 34.65

Table S3. Denitrification and nitrification rates (ng $L^{-1}h^{-1}$) from Fig. 6 in Ref. [1].

	Field measurements	Experiment rate in light	Experiment rate in dark
		ng $L^{-1}h^{-1}$	
Denitrification	3.3	6.5	7.5
Nitrification	1.6	0	2.3

Table S4. Contribution of nitrification and denitrification to the nitrate pool (tonnes N summer^{-1} or tonnes N y^{-1}) in Kongsfjorden based on field measurements and on laboratory incubations. Denitrification is not sensitive to light, but nitrification is only active in the dark. To calculate nitrification, we consider only the number of hours without daylight in summer and per year. Water depths below 50 m are assumed to be in the dark.

	**Year**		**Summer (July and August)**	
	Based on field measurements	Based on incubation experiments	Based on field measurements	Based on incubation experiments
Surface (0 - 50 m)				
Denitrification	280	595	48	101
Nitrification	64	93	0.3	0.4
Net contribution	-215	-501	-47	-101
Deep layer (50 m to seafloor)				
Denitrification	526	1115	89	189
Nitrification	255	366	43	62
Net contribution	-271	-748	-46	-127
Total (0 m - seafloor)				
Denitrification	806	1710	137	290
Nitrification	319	459	43	62

Net contribution	-487	-1251	-94	-228

Table S5. Coefficients for the piecewise polynomial function (PCHIP) to fit the biomass along the depth gradient.

**Interval: $x_1 - x_2$ (m)**	**Coefficients × 10^4**			
	**a**	**b**	**c**	**d**
0 - 2.5	-0.0058	-0.1636	0.9269	0.1908
2.5 - 5	0.1119	-0.4433	0	1.3949
5 - 10	-0.0005	0.0137	-0.1185	0.3727
10 - 15	0.0001	0.0009	-0.0198	0.0588
15 - 20	-0.0000	0.0000	0.000	0.000
20 - 50	-0.0000	-0.0000	0.000	0.000

Equation form: $f(x) = a(x_2-x_1)^3 + b(x_2-x_1)^2 + c(x_2-x_1) + d$

Table S6. Biogeochemical ammonium *Sources-Sinks* estimated with equation 1, based on average salinities and concentrations of ammonium in AW found in the shelf stations V10, V12, and V14, in the top 100 m, and comparable values in IW, SW or AW found in the fjord stations Kb1-Kb5, and using 1.44 µmol kg^{-1} for the freshwater endmember.

Ammonium *Sources-Sinks* (µmol kg^{-1})			
**Year**	**AWs *versus* IW**	**AWs *versus* SW**	**AWs *versus* AWf**
2013	0.9	0.10	1.6
2014	0.8	0.43	2.0
2015	0.0	-0.3	0.7
2016	0.9	0.98	1.8
2017	0.7	0.43	1.4
2020	0.9	0.65	1.3

Footnote: Refer to Methodology - Freshwater inputs, for nutrient concentrations in freshwater and Table S2 for water-mass classification. Subscripts s and f are used to distinguish AW in the shelf and in the fjord, respectively. Values in bold correspond to significant concentration differences between the water masses (two-tailed t-test, $p < 0.05$).

Table S7. Biogeochemical phosphate *Sources-Sinks* estimated with equation 1, based on average salinities and concentrations of phosphate in AW found in the shelf stations V10, V12, and V14, in the top 100 m, and comparable values in IW, SW or AW found in the fjord stations Kb1-Kb5, and using 0.064 μmol kg^{-1} for the freshwater endmember.

Phosphate *Sources-Sinks* (μmol kg^{-1})			
**Year**	**AWs *versus* IW**	**AWs *versus* SW**	**AWs *versus* AWf**
2011	-0.2	-0.1	-
2012	-0.2	-0.3	-0.2
2013	-0.2	-0.2	-0.1
2014	-0.3	0.0	0.0
2015	-0.2	-0.6	-0.3
2016	-0.6	-0.6	-0.3
2017	-0.1	-0.2	0.1
2018	-0.1	-0.1	0.2
2019	-0.2	-0.2	-0.1
2020	-0.2	-0.3	0.0

Footnote: Refer to Methodology - Freshwater inputs, for nutrient concentrations in freshwater and Table S2 for water-mass classification. Subscripts s and f are used to distinguish AW in the shelf and in the fjord, respectively. No data is marked by the dash "-" symbol. Values in bold correspond to significant concentration differences between the water masses (two-tailed t-test, $p < 0.05$).

Table S8. Biogeochemical silicic acid *Sources-Sinks* estimated with equation 1, based on average salinities and concentrations of silicic acid in AW found in the shelf stations V10, V12, and V14, in the top 100 m, and comparable values in IW, SW or AW found in the fjord stations Kb1-Kb5, and using 9.77 µmol kg^{-1} for the freshwater endmember.

**Silicic acid *Sources-Sinks* (µmol kg^{-1})**			
**Year**	**AWs *versus* IW**	**AWs *versus* SW**	**AWs *versus* AWf**
2011	-0.9	-1.5	-
2012	-2.2	-2.5	-2.3
2013	-0.4	-1.1	-0.2
2014	-1.6	-1.3	-0.6
2015	-2.1	-2.5	-0.5
2016	-1.8	-1.7	-0.4
2017	-1.0	-0.9	0.8
2018	-1.5	-1.5	0.0
2019	-1.6	-1.1	-0.2
2020	-1.4	-1.8	-0.2

Footnote: Refer to Methodology - Freshwater inputs, for nutrient concentrations in freshwater and Table S2 for water-mass classification. Subscripts s and f are used to distinguish AW in the shelf and in the fjord, respectively. No data is marked by the dash "-" symbol. Values in bold correspond to significant concentration differences between the water masses (two-tailed t-test, $p < 0.05$).

Table S9. Biogeochemical nitrate + nitrite *Sources-Sinks* estimated with equation 2, based on average salinities and concentrations of nitrate + nitrite in AW found in the shelf stations V10, V12 and V14, in the top **50 and 150 m**, and comparable values in IW, SW or AW, found in the fjord stations Kb1-Kb5. These estimates assume 2.0 µmol kg^{-1} for the freshwater endmember. No data is marked by the dash "-" symbol. Refer to paper Methodology- Freshwater inputs for nutrient concentrations in freshwater and Table S2 for water mass classification.

Nitrate + nitrite *Sources-Sinks* (µmol kg^{-1})						
	**Estimates based on top 50 m**			**Estimates based on top 150 m**		
**Year**	**AW *versus* IW**	**AW *versus* SW**	**AW *versus* AW**	**AW *versus* IW**	**AW *versus* SW**	**AW *versus* AW**
2011	-2.0	-1.2	-	-2.0	-1.2	-
2012	-3.8	-4.0	-4.1	-4.9	-5.0	-5.2
2013	-1.8	-2.4	-0.7	-3.7	-4.2	-2.6
2014	-3.7	-3.1	-3.2	-4.7	-4.0	-2.6
2015	-4.9	-5.2	-2.4	-7.5	-7.7	-4.4
2016	-4.3	-4.1	-3.2	-5.3	-5.0	-2.5
2017	-2.4	-2.3	-0.8	-3.3	-3.2	0.5
2018	-0.5	-0.7	-	-1.7	-1.8	1.9
2019	-2.8	-2.6	-2.6	-4.0	-3.7	-2.9
2020	-3.3	-3.9	-2.5	-4.7	-5.2	-1.8

Figures S1 to S21

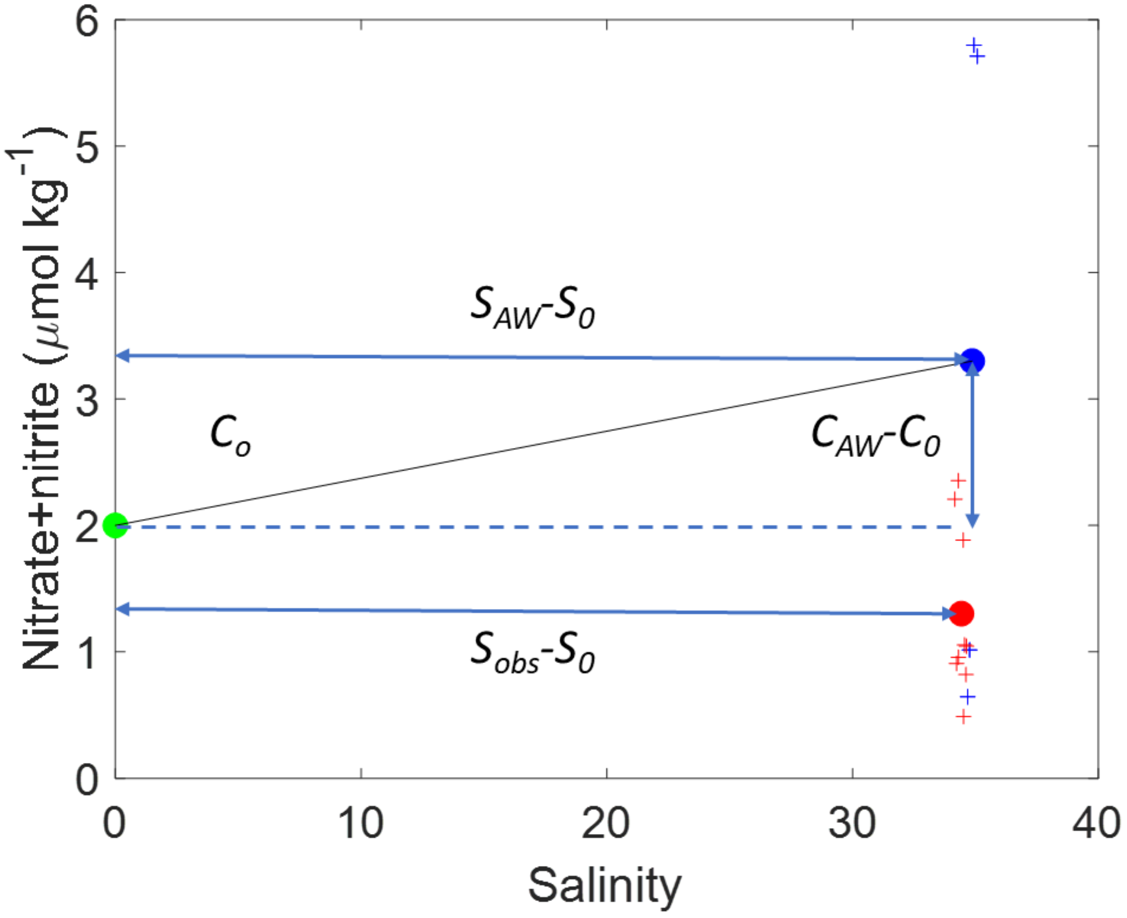

Figure S1. Example of a nutrient-salinity diagram including the concentration at the freshwater endmembers (green dots at zero salinity), in the marine endmember [Atlantic Water (AW), blue crosses and dots] and, in Intermediate Water (IW, red crosses and dots) inside the fjord. Dots are arithmetic averages of the data represented by the crosses of the same color. These averages were used in equation 1 for the various terms. The symbology used here is the same as in equation 1. Refer Table S2 for the definition of the various water masses.

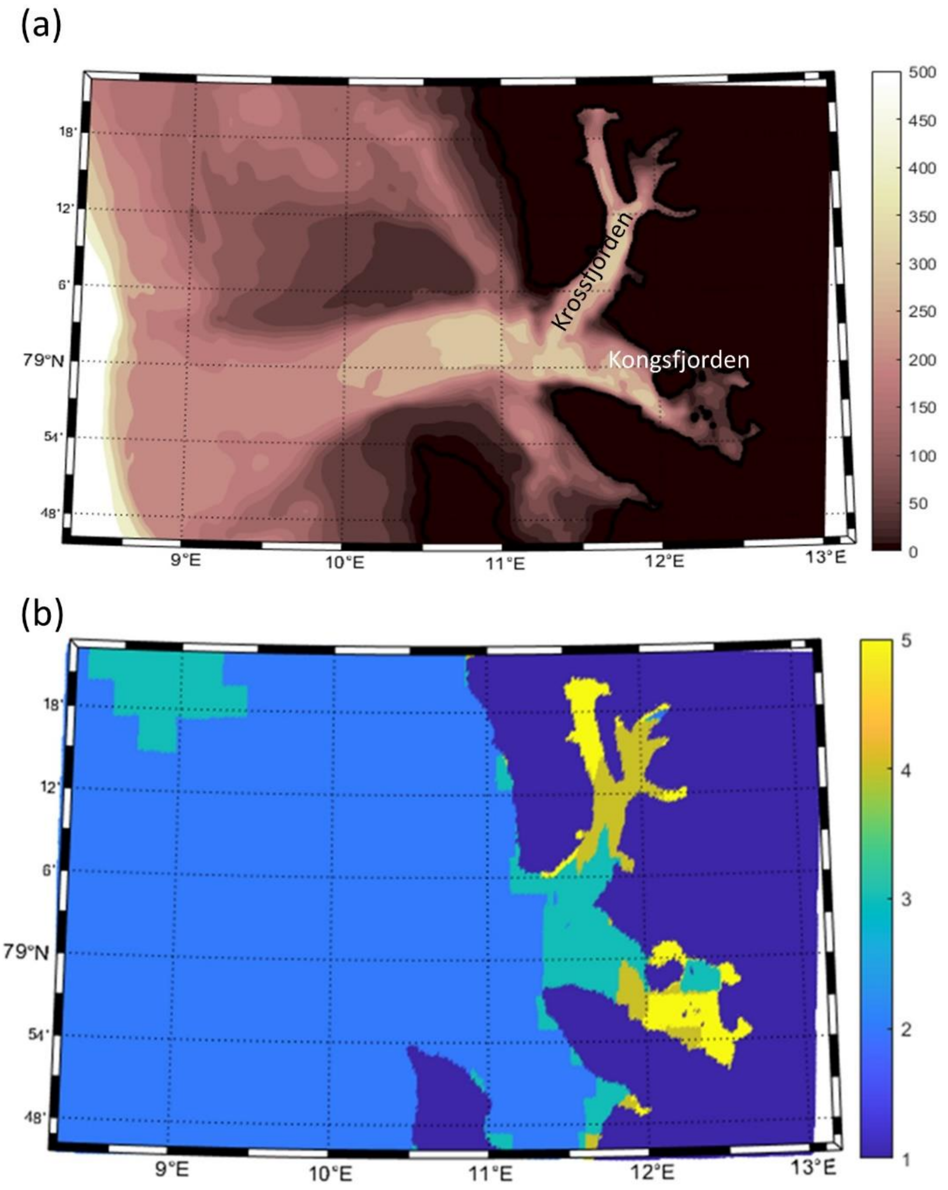

Figure S2. (a) K160_bgc model domain and bathymetry [color scale corresponding to depths in meters (m)], showing Kongsfjorden, Krossfjorden and the adjacent coastal area; (b) Jerlov water types used in this study: 1, 2, 3, 4 and 5 correspond to types I, IA, IB, II and III [e.g. Ref. [50]].

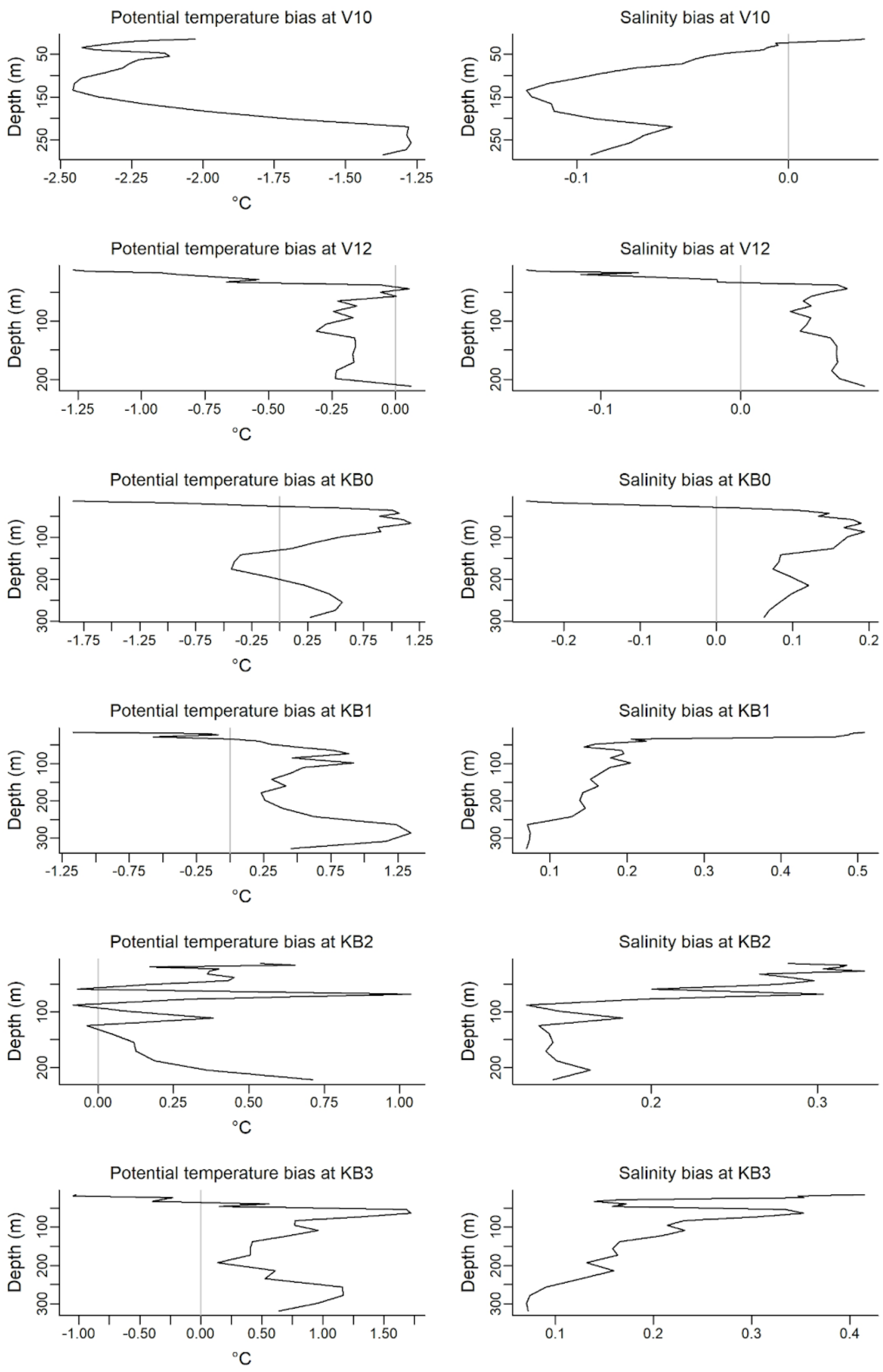

Figure S3. Model temperature and salinity biases from CTD profiles for stations shown in Fig. 1 in the main text. Only depths greater than 10 m are considered.

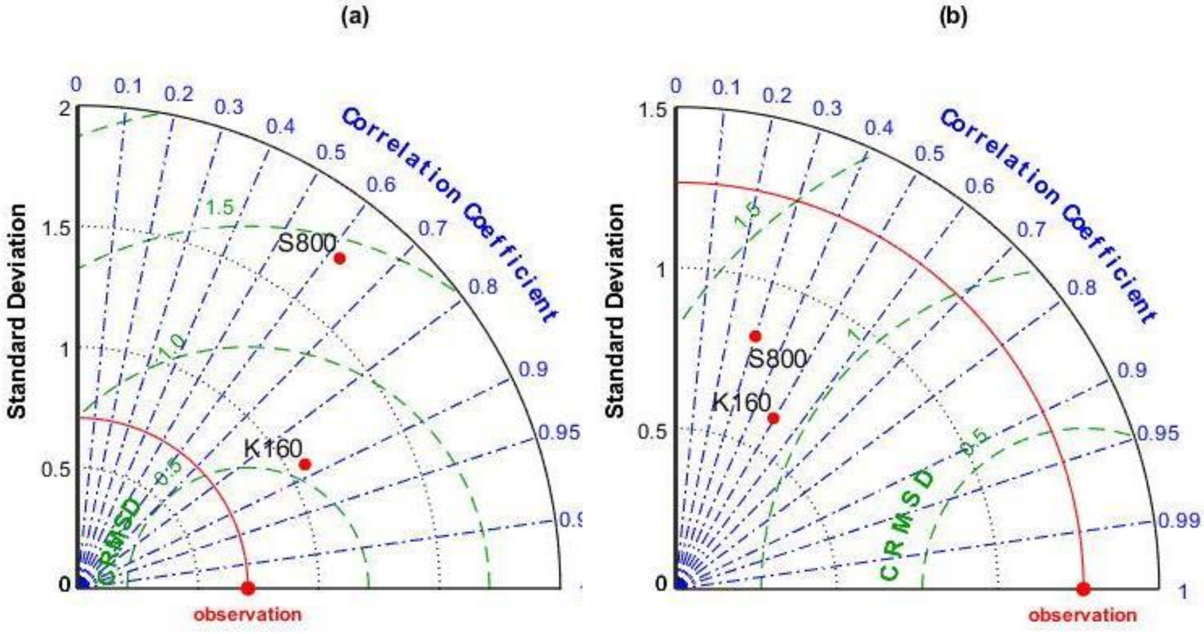

Figure S4. Taylor diagrams based on results from >300 CTD profiles collected in 2007 for salinity (a) and temperature (b), and results from the S800 and the K160 models, where the boundaries of the latter are interpolated from the results of the former model. The red line depicts the standard deviation of the observations. CRMSD stands for Centered Root Mean Square Difference.

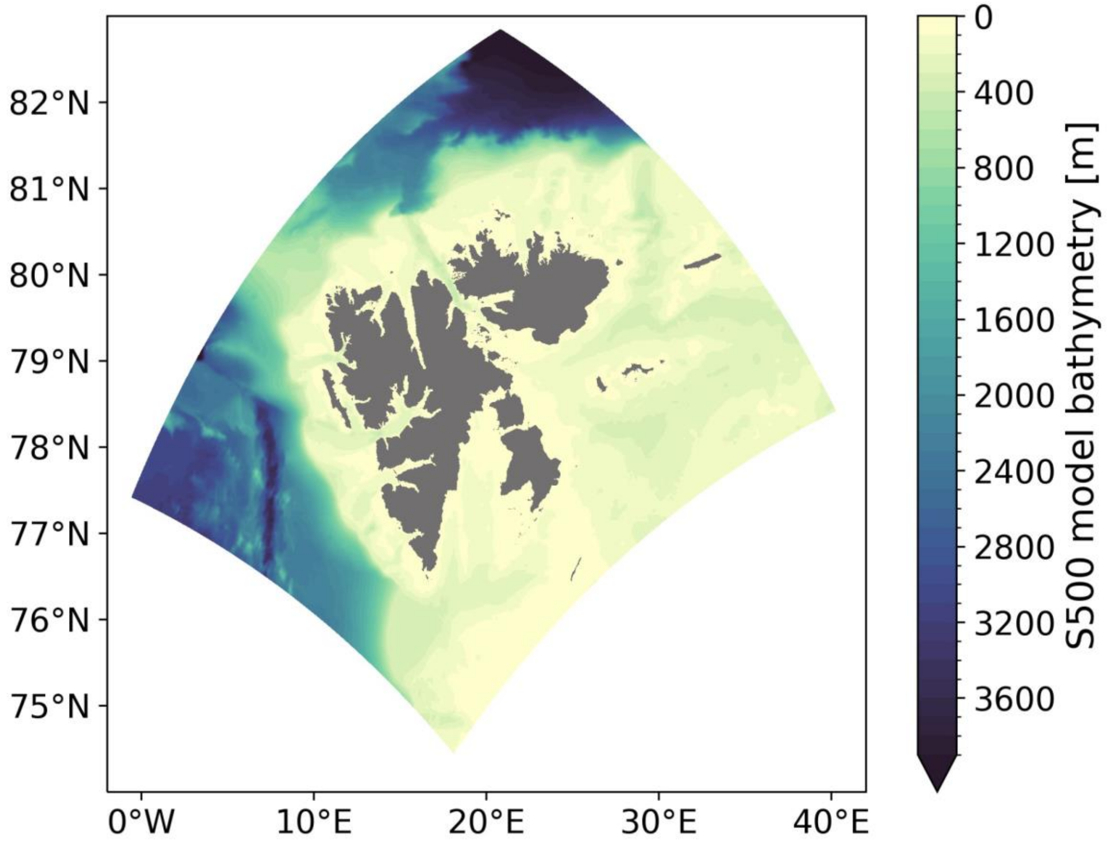

Figure S5. Domain and bathymetry of the S500 model used to provide boundary conditions for the K160_bgc model (2019 simulations).

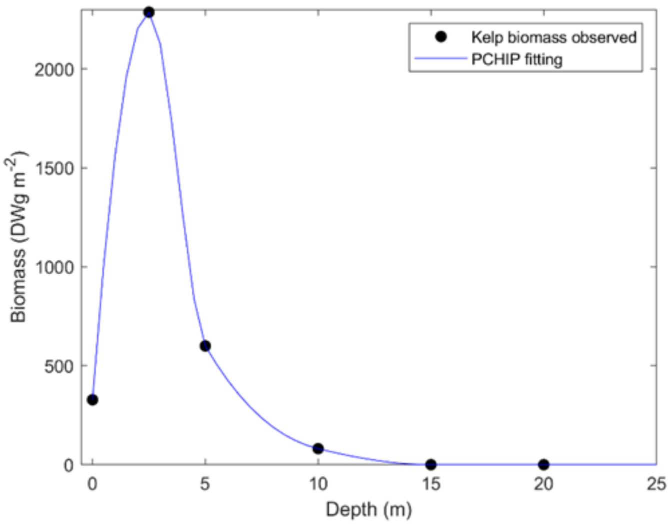

Figure S6. Distribution of kelp biomass along a depth gradient at Hansneset, Kongsfjorden. We forced the biomass at 20 and 50 m to be 0 to maintain low biomass values at depth.

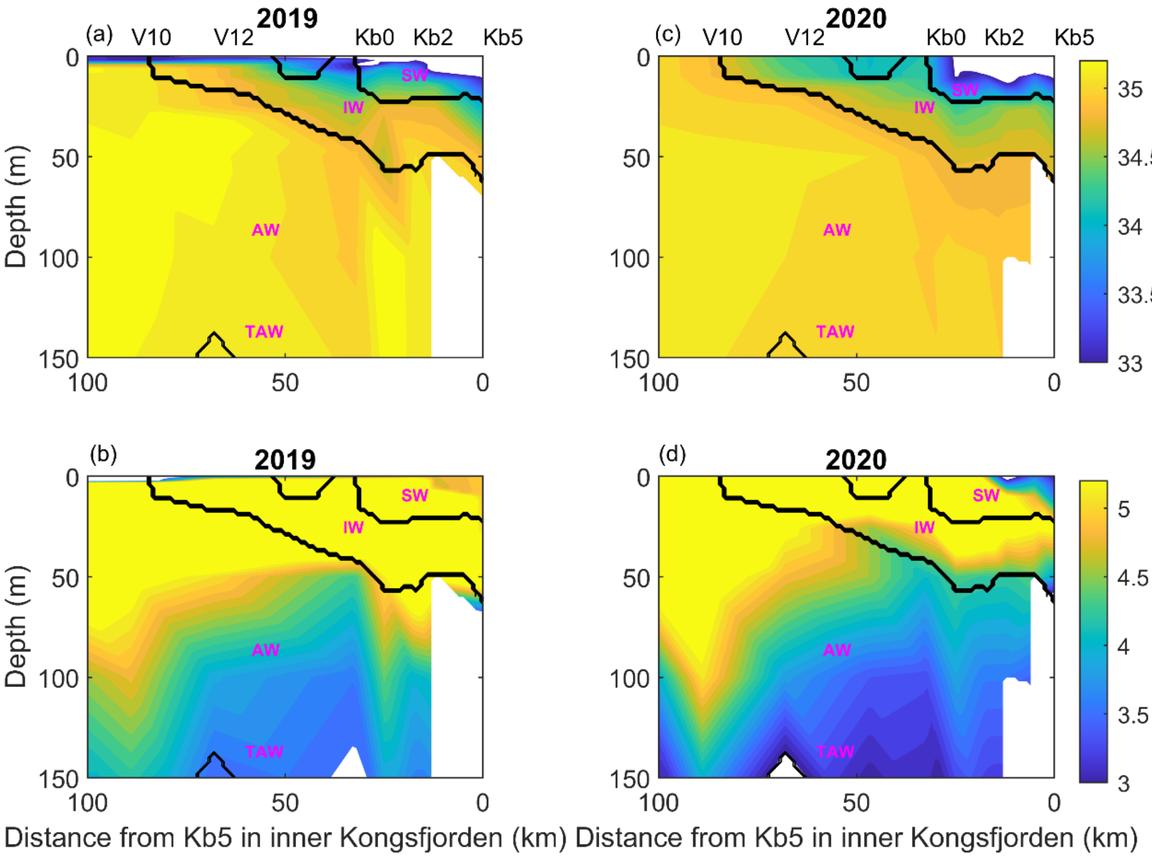

Figure S7. Contours of absolute salinity (top) and conservative temperature (bottom) for 2019 (a-b) and 2020 (c-d) from the MOSJ dataset for the Kongsfjorden summer transect, and water masses delimited by the black lines, according to [49] (AW – Atlantic Water, TAW – Transformed Atlantic Water, SW – Surface Water, and IW – Intermediate Water). A subset of the sampling stations is plotted over panels a, c. Refer to Table S2 for water mass characteristics and Fig. 1b, c for the location of all sampling stations. The white areas in the contour plots correspond to places where data were not available. In the case of stations Kb2 and Kb5 there were no data below 100 m due to depth constraints.

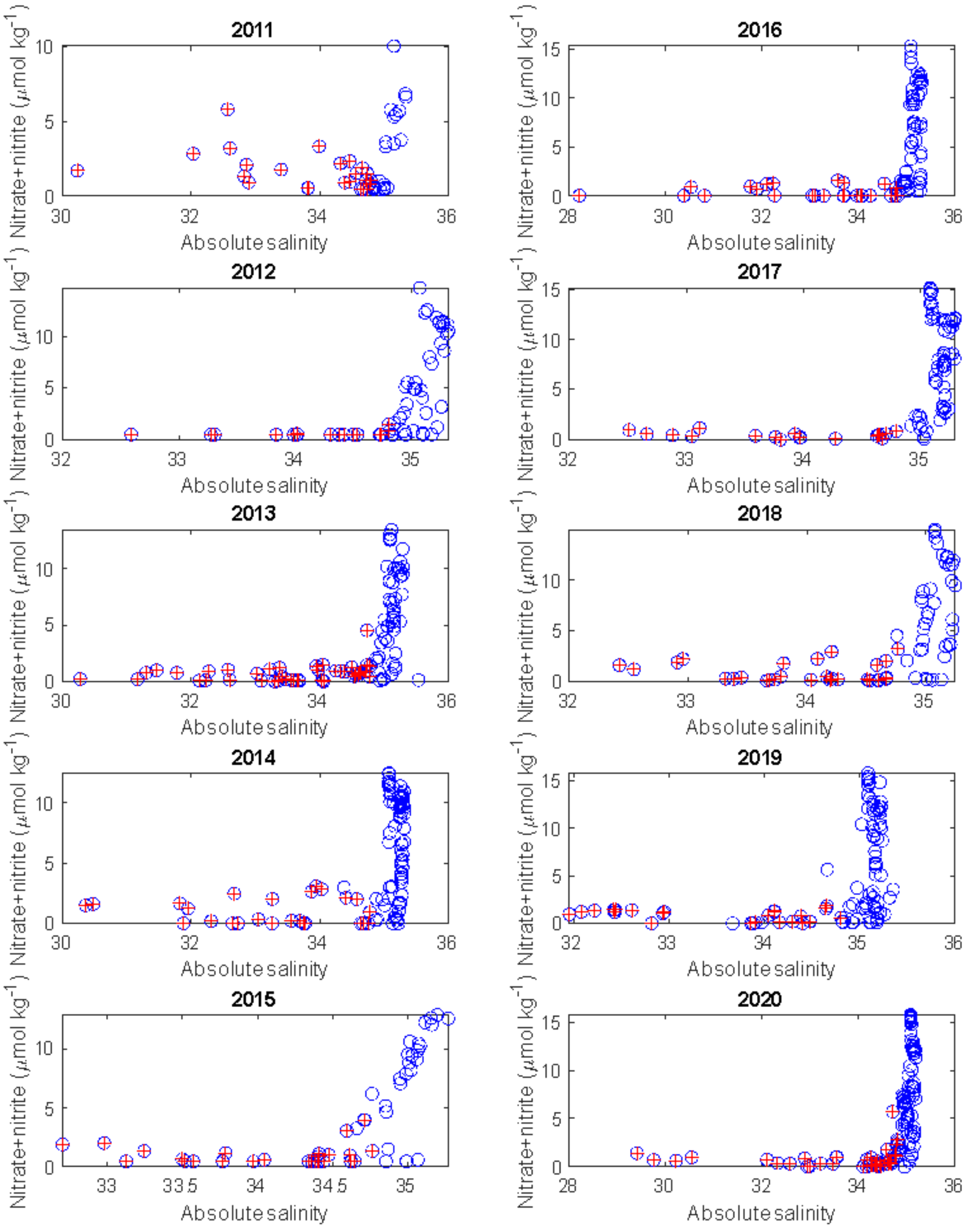

Figure S8. Nitrate + nitrite-salinity diagrams for the whole sampling period based on summer MOSJ data. Blue circles contain data from all water masses found in the study area, whereas red crosses are only for SW – Surface Water, and IW – Intermediate Water (refer to Table S2 for water mass characteristics).

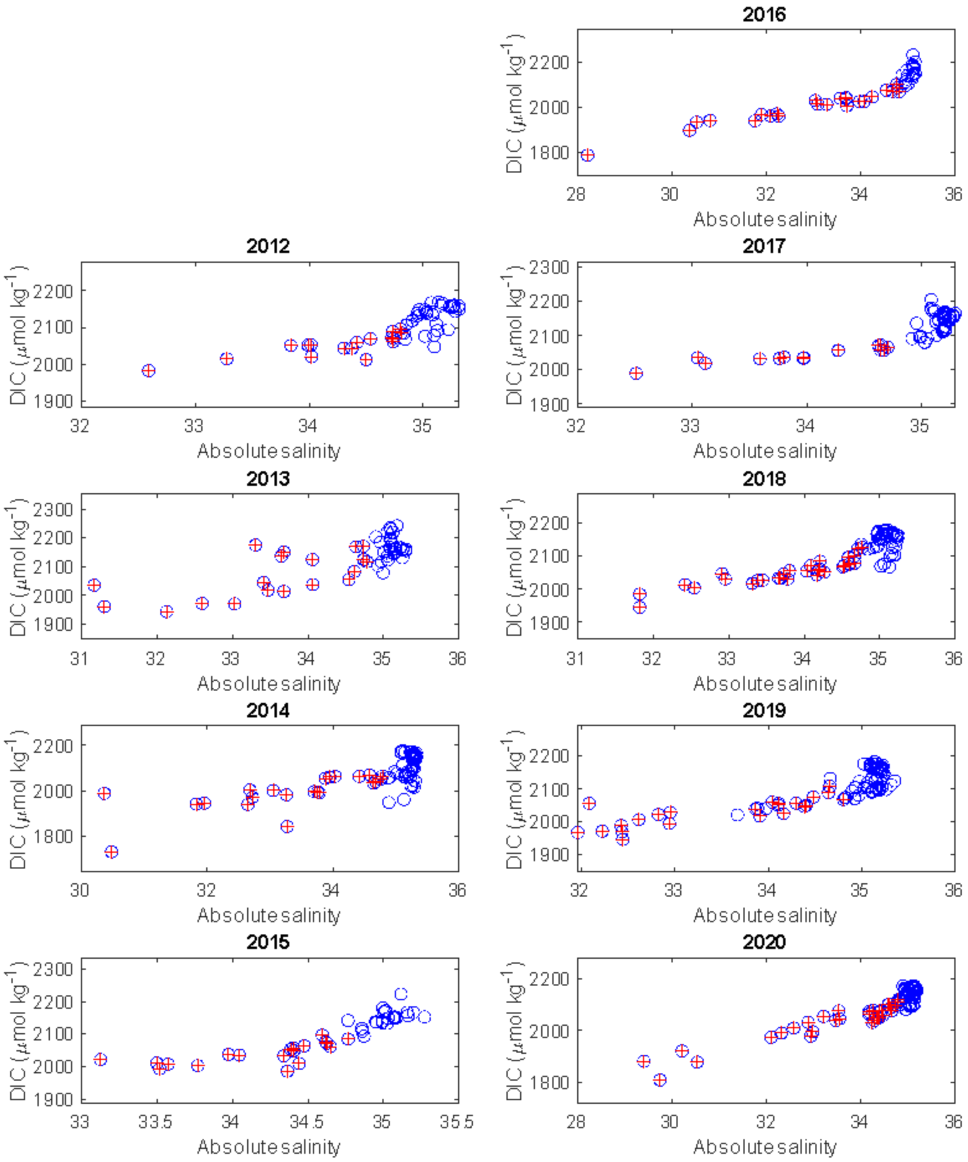

Figure S9. Dissolved inorganic carbon-salinity diagrams for the whole sampling period based on summer MOSJ data. Blue circles contain data from all water masses found in the study area, whereas red crosses are only for SW – Surface Water, and IW – Intermediate Water (refer to Table S2 for water mass characteristics).

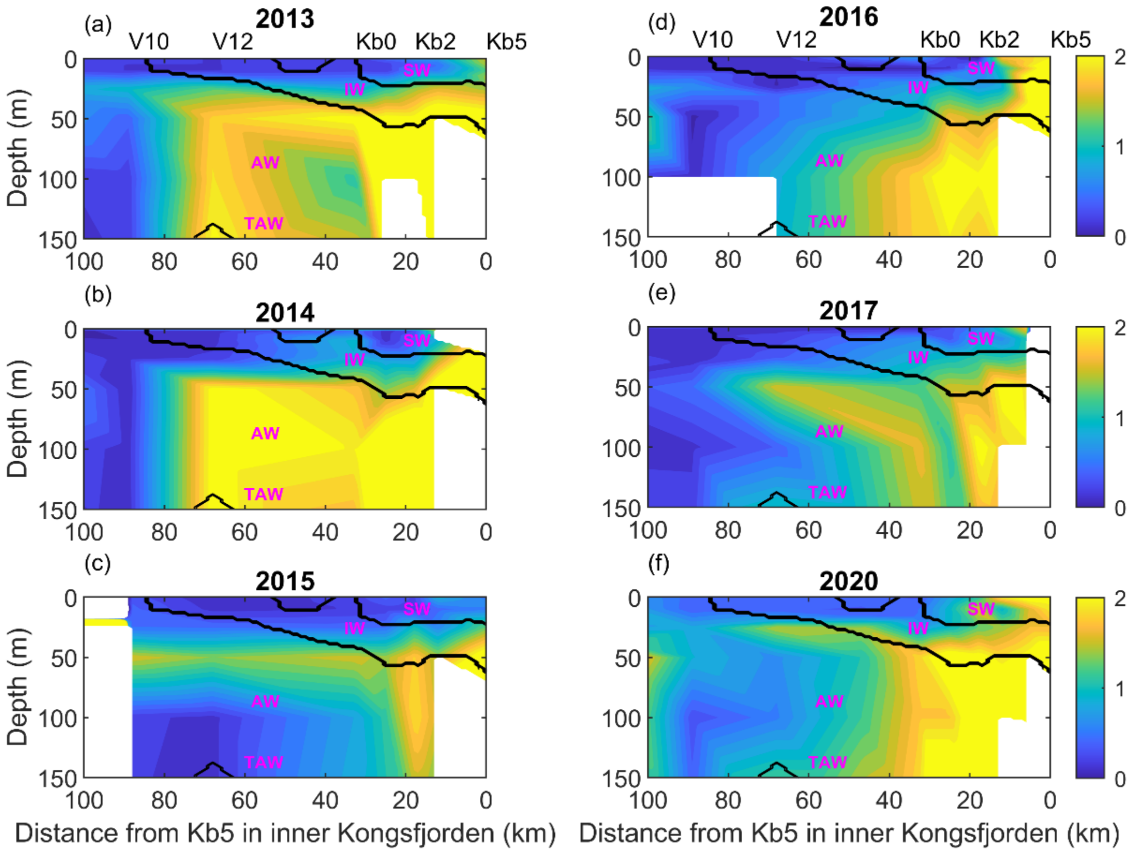

Figure S10. Ammonia concentration contours (µmol kg^{-1}) for 2013-2017 and 2020 based on MOSJ dataset for the Kongsfjorden summer transect, and water masses delimited by the black lines, according to [49] (AW – Atlantic Water, TAW – Transformed Atlantic Water, SW – Surface Water, and IW – Intermediate Water). A subset of the sampling stations is plotted over panels a, d. Refer to Table S2 for water mass characteristics and Figs. 1b, c for the location of all sampling stations. The white areas in the contour plots correspond to places where data were not available. In the case of stations Kb2 and Kb5 there were no data below 100 m due to depth constraints.

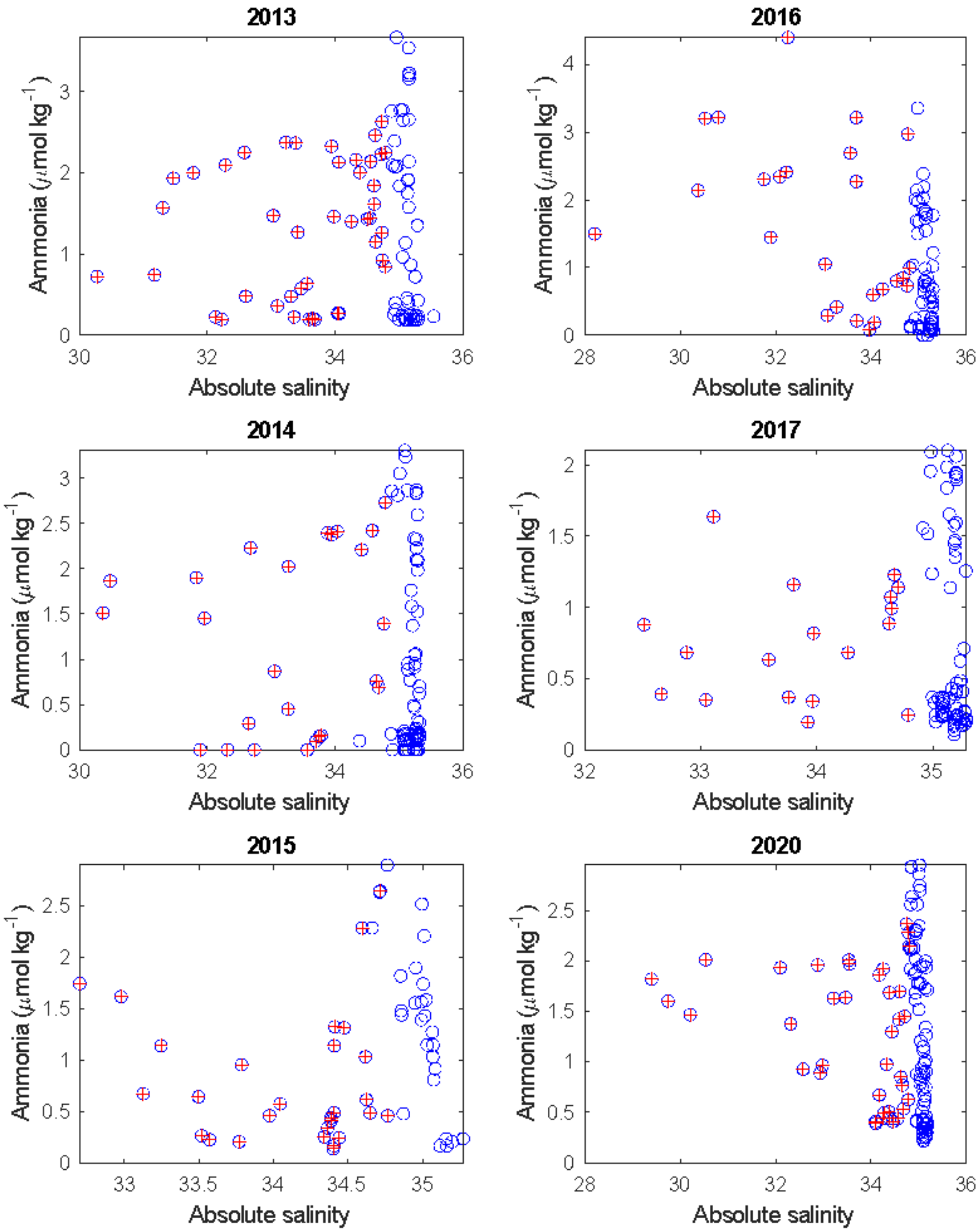

Figure S11. Ammonia-salinity diagrams for the whole sampling period based on summer MOSJ data. Blue circles contain data from all water masses found in the study area, whereas red crosses are only for SW – Surface Water, and IW – Intermediate Water (refer to Table S2 for water mass characteristics).

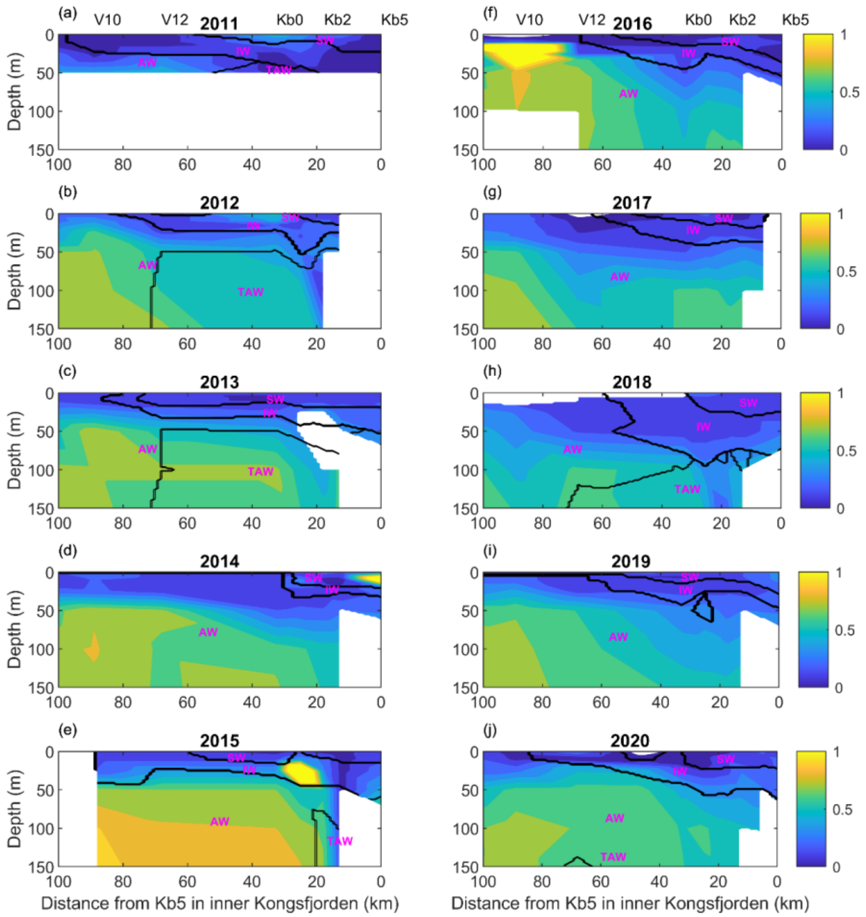

Figure S12. Phosphate concentration contours (μmol kg^{-1}) for 2011 - 2020 based on MOSJ dataset for the Kongsfjorden summer transect, and water masses delimited by the black lines, according to [49] (AW – Atlantic Water, TAW – Transformed Atlantic Water, SW – Surface Water, and IW – Intermediate Water). A subset of the sampling stations is plotted over panels a, f. Refer to Table S2 for water mass characteristics and Figs. 1b, c for the location of all sampling stations. plotted over panels (a) and (f). Refer to Table S2 for water mass characteristics and Figs. 1c, d for the location of all sampling stations. The white areas in the contour plots correspond to places where data were not available. In the case of stations Kb2 and Kb5 there were no data below 100 m due to depth constraints.

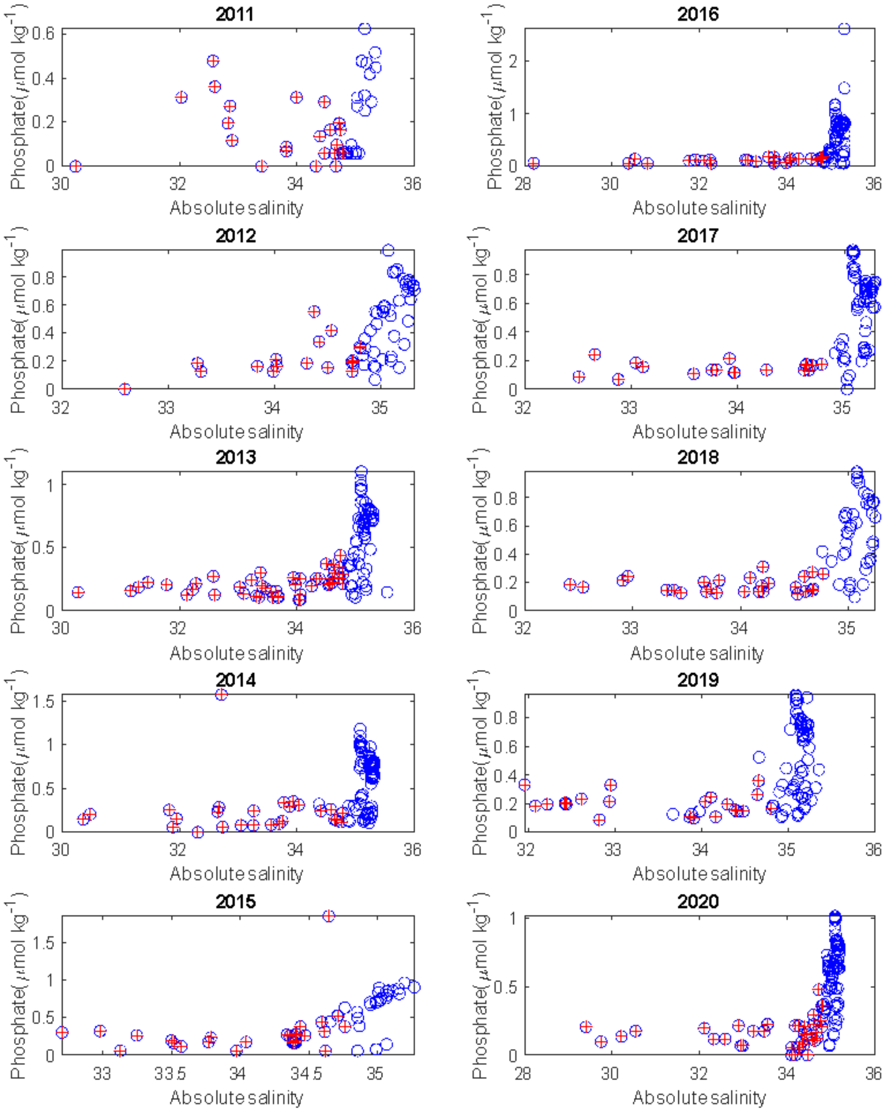

Figure S13. Phosphate-salinity diagrams for the whole sampling period based on summer MOSJ data. Blue circles contain data from all water masses found in the study area, whereas red crosses are only for SW – Surface Water, and IW – Intermediate Water (refer to Table S2 for water mass characteristics).

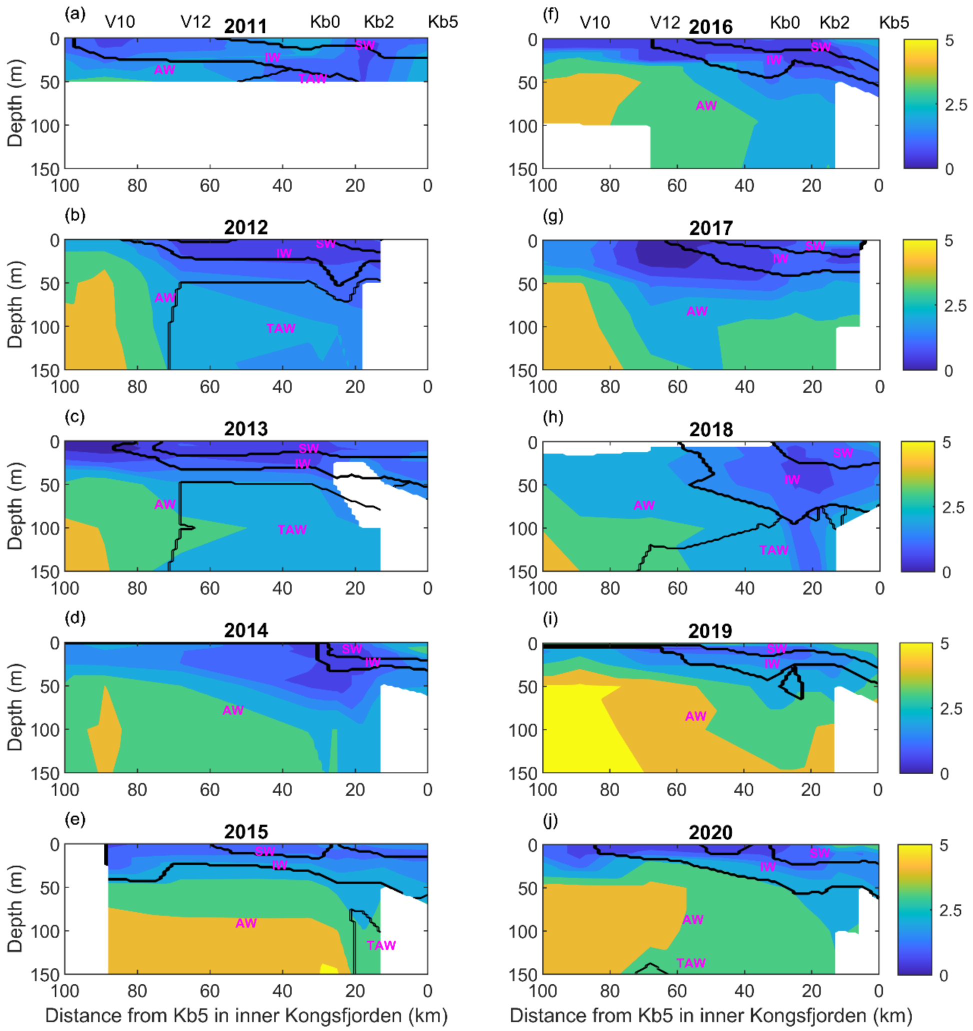

Figure S14. Silicic acid concentration contours (μmol kg^{-1}) for 2011 - 2020 based on MOSJ dataset for the Kongsfjorden summer transect, and water masses delimited by the black lines, according to [49] (AW – Atlantic Water, TAW – Transformed Atlantic Water, SW – Surface Water, and IW – Intermediate Water). A subset of the sampling stations is plotted over panels a, f. Refer to Table S2 for water mass characteristics and Figs. 1b, c for the location of all sampling stations. The white areas in the contour plots correspond to places where data were not available. In the case of stations Kb2 and Kb5 there were no data below 100 m due to depth constraints.

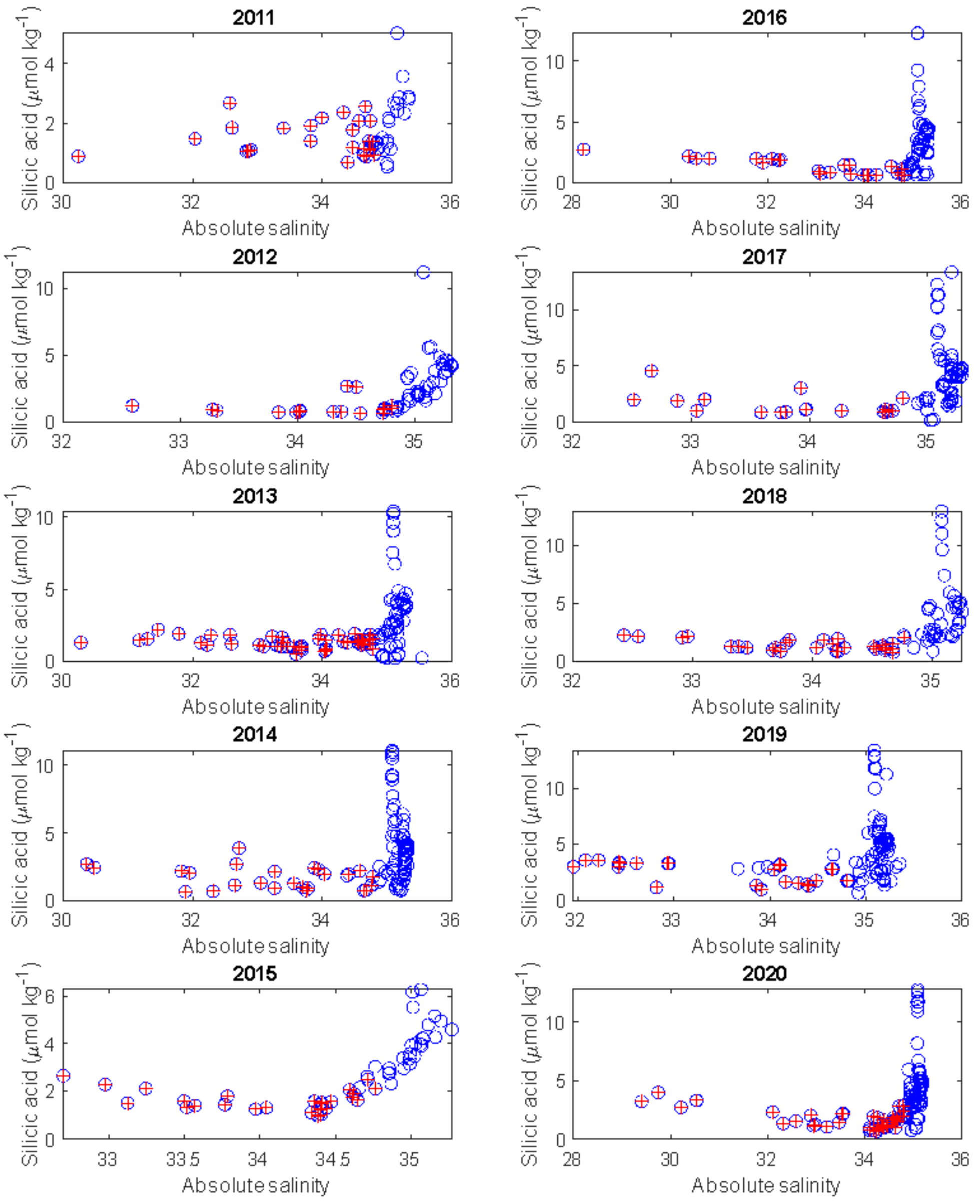

Figure 15. Silicic acid -salinity diagrams for the whole sampling period based on summer MOSJ data. Blue circles contain data from all water masses found in the study area, whereas red crosses are only for SW – Surface Water, and IW – Intermediate Water (refer to Table S2 for water mass characteristics).

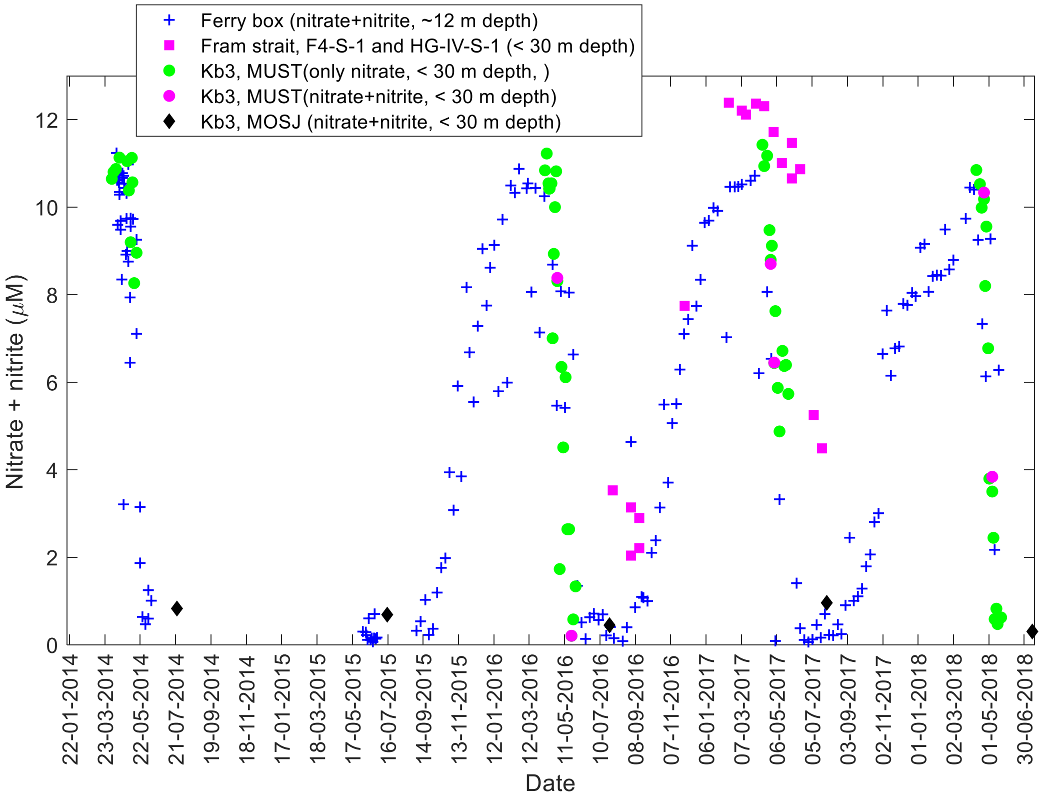

Figure S16. Nitrate + nitrite data from the MOSJ, AMUST and Torres-Valdes et al. [Ref. [46]] datasets for stations outside Kongsfjorden (F4-S-1 and HG-IV-S-1, magenta squares) and inside Kongsfjorden (Kb3) and from the AWIPEV dataset (Ferry Box, also inside Kongsfjorden and close to Kb3), all the other symbols (refer Table S1 and Fig. 1). Concentration shown in μM instead of μmol L^{-1} due to the lack of salinity data for some of the datasets.

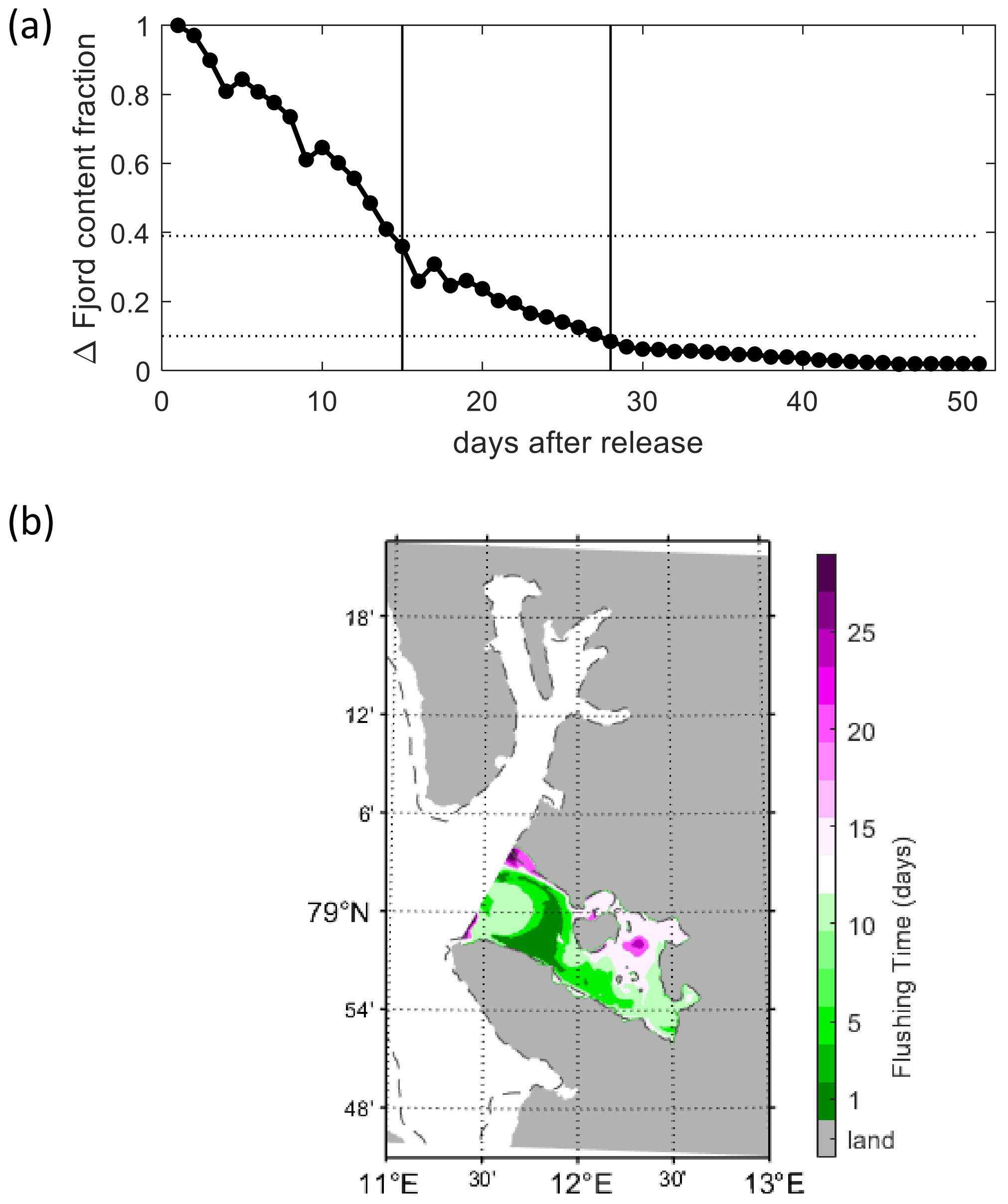

Figure S17. Results from a simulation between 01.06.2007 and 31.07.2007. (a) Change in the tracer contents within the fjord relative to day 1 of the simulation, calculated with equation S2. Black vertical lines mark the time needed for the flushing of 60 and 90% of a tracer (~15 and ~28 days, respectively). The first threshold was used as proxy to the fjord flushing time. (b) Spatial variability of flushing time of surface water (0-100 m) in Kongsfjorden.

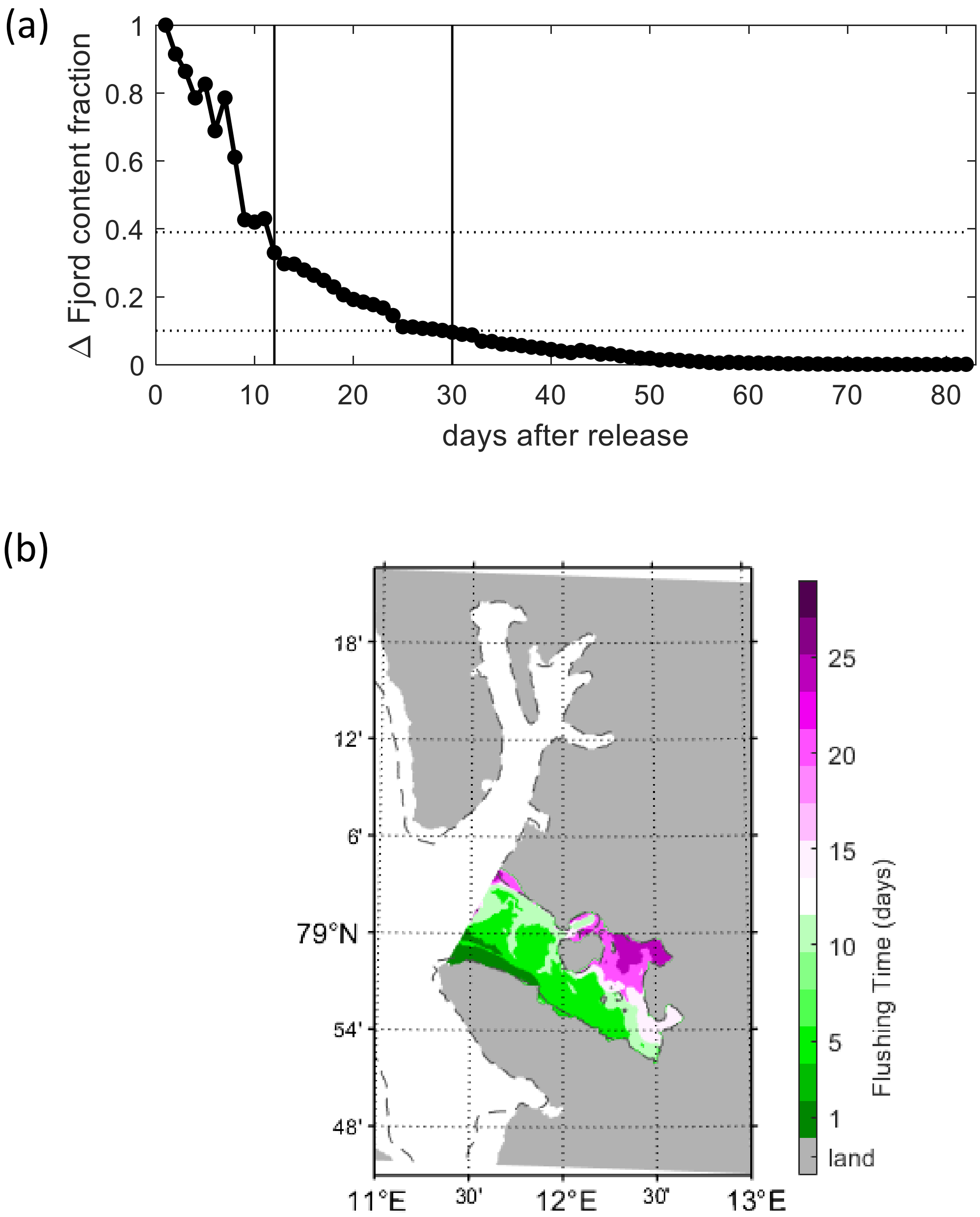

Figure S18. Results from a simulation between 01.04.2019 and 23.06.2019. (a) Change in the tracer contents within the fjord relative to day 1 of the simulation, calculated with equation S2. Black vertical line marks the time needed for the flushing of 60 and 90% of a tracer (~10 and ~30 days, respectively). The first threshold was used as proxy to the fjord flushing time. (b) Spatial variability of flushing time of surface water (0-100 m) in Kongsfjorden.

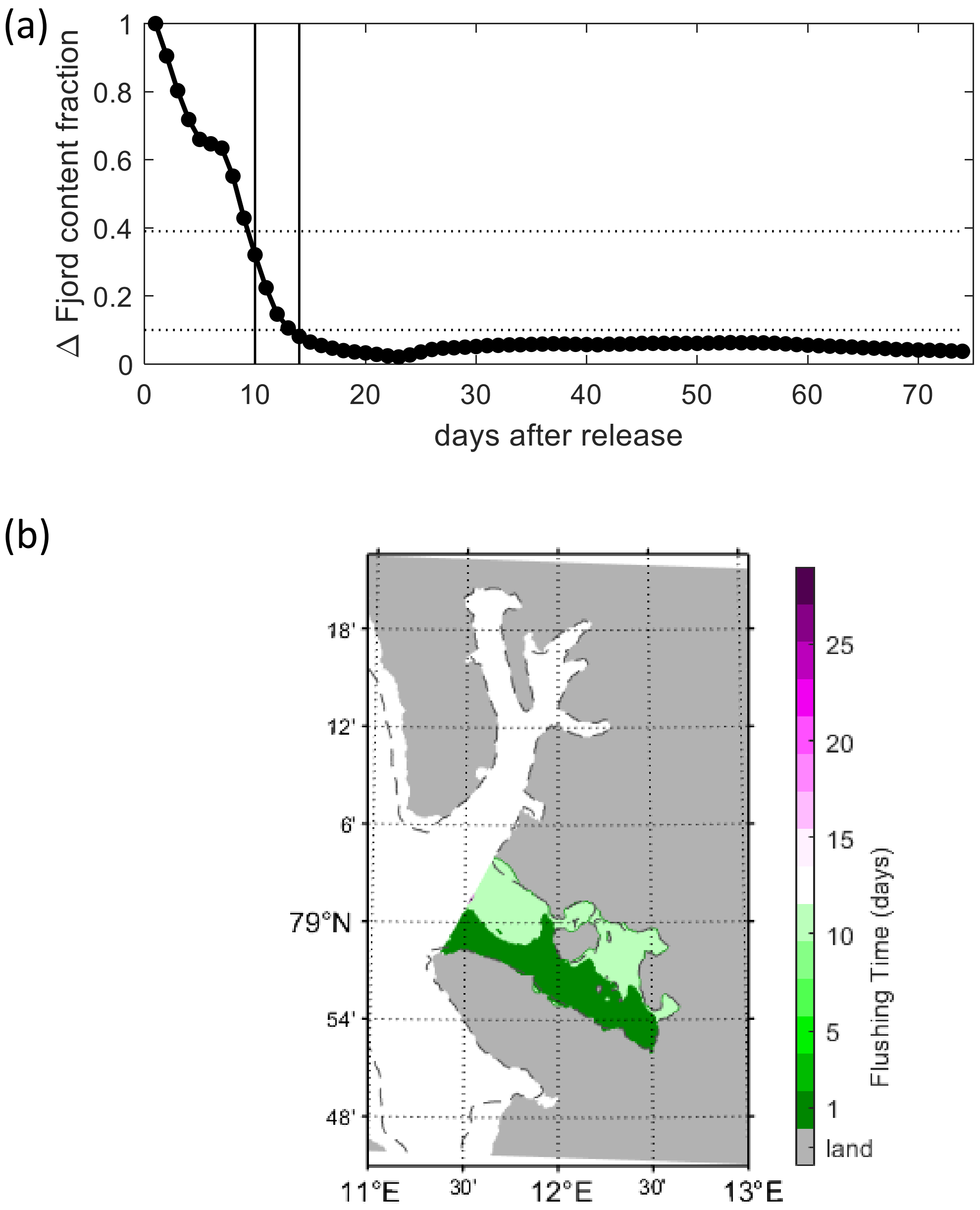

Figure S19. Results from a simulation between 23.06.2019 and 05.09.2019. (a) Change in the tracer contents within the fjord relative to day 1 of the simulation, calculated with equation S2. Black vertical line marks the time needed for the flushing of 60 and 90% of a tracer (~10 and ~12 days, respectively). The first threshold was used as proxy to the fjord flushing time. (b) Spatial variability of flushing time of surface water (0-100 m) in Kongsfjorden.

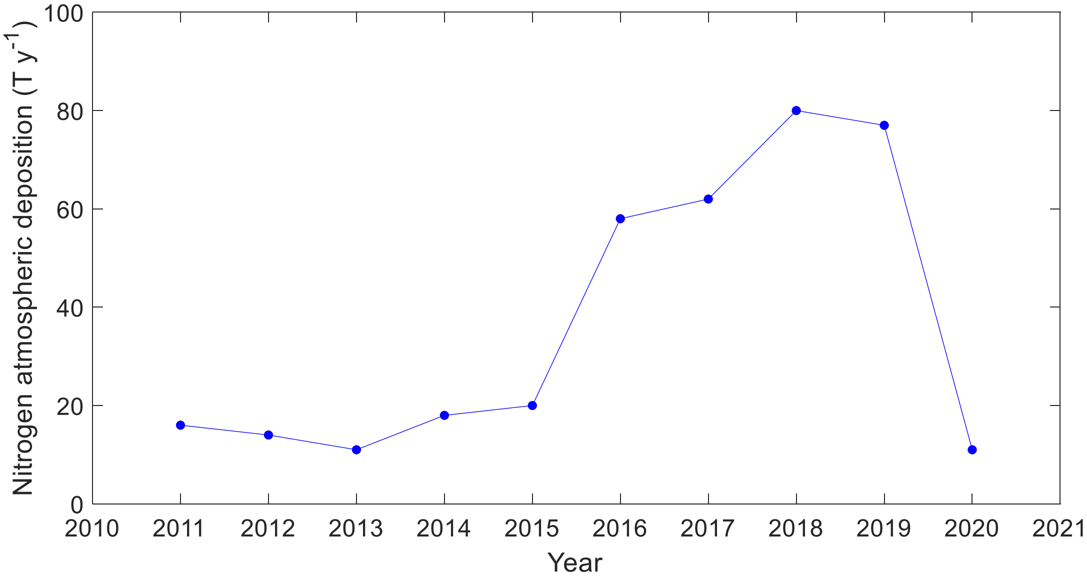

Figure S20. Yearly averaged atmospheric nitrogen loads (see text).

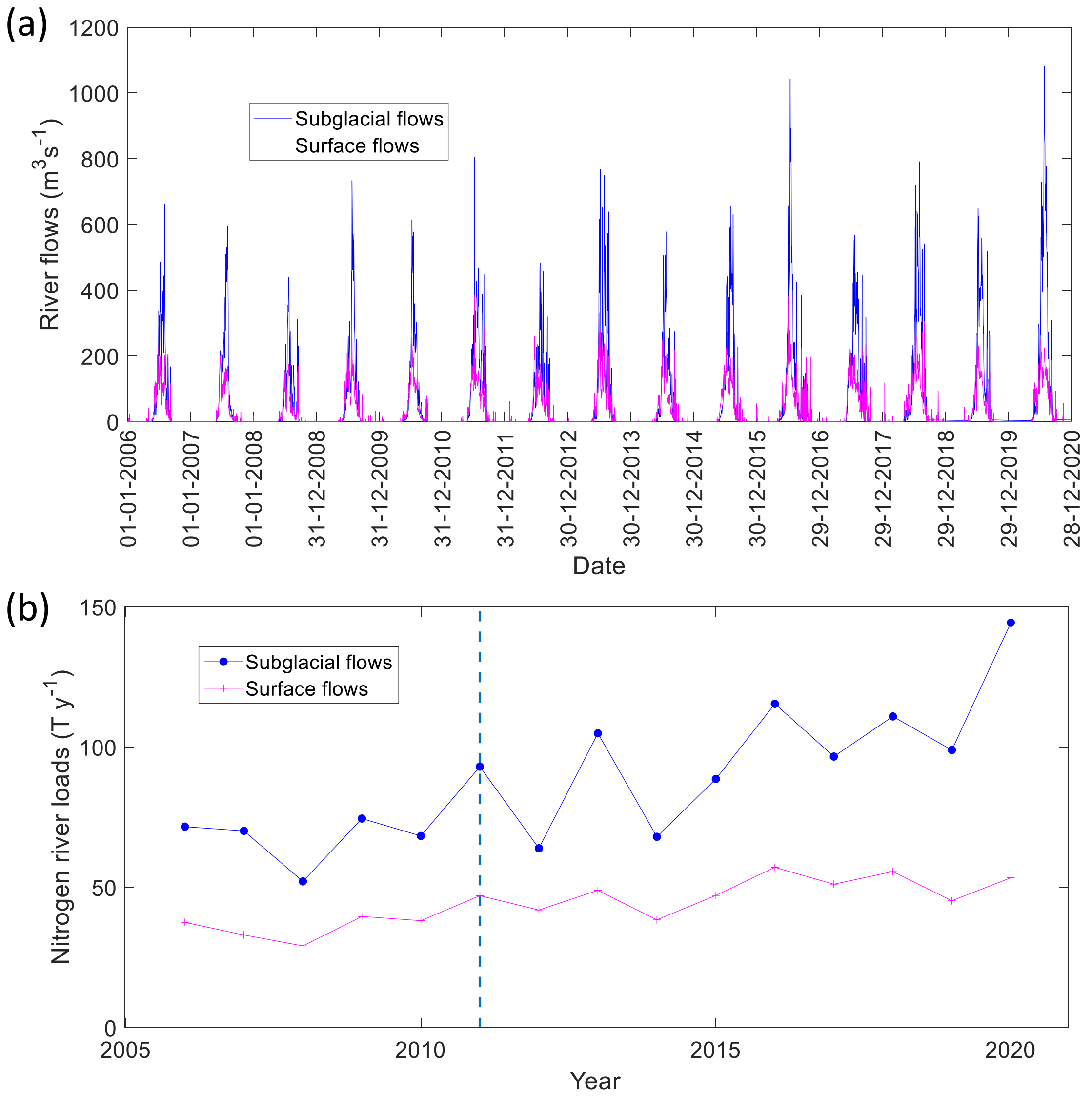

Figure S21. (a) Daily subglacial and surface glacier river flows from the model of van Pelt et al. [Ref. [40]]; (b) Yearly averaged nitrogen loads with the river flows. The dashed line marks the year of 2011, which is the beginning of our study period (see text).

References

1. Krishnan, K. P., Sinha, R. K. & Rajan, S. Pelagic nitrification and denitrification rates in an Arctic fjord during early spring. *Ann. Microbiol.* **64,** 365-376;10.1007/s13213-013-0671-4 (2014).
2. Pavlov, A. K. et al. The underwater light climate in Kongsfjorden and its ecological implications in *The Ecosystem of Kongsfjorden, Svalbard*, (eds. Hop, H. & Wiencke, W.). *Advances in Polar Ecology*, **Vol. 2,** 137-172**.** Springer, Cham;10.1007/978-3-319-46425-1_5 (2019).
3. Niedzwiedz, S. & Bischof, K. Glacial retreat and rising temperatures are limiting the expansion of temperate kelp species in the future Arctic. *Limnol. Oceanogr.* **68,** 816-830; 10.1002/lno.12312 (2023).
4. Hop, H. et al. The marine ecosystem of Kongsfjorden, Svalbard. *Polar Res.* **21,** 167-208; 10.1111/j.1751-8369.2002.tb00073.x (2002).
5. Wold, A. et al. Atlantification influences zooplankton communities seasonally in the northern Barents Sea and Arctic Ocean. *Prog. Oceanogr.* **219**: 103133. doi: 10.1016/J.Pocean.2023.103133 (2023).
6. Jørgensen, S. E., Nielsen, S. N. & Jørgensen, L. A. Handbook of ecological parameters and ecotoxicology. Elsevier (1991).
7. Gentili, B., R. K. Singh, S. Bélanger, R. Schlegel & Gattuso, J-P. FjordLight: PAR data for Arctic fjords. PANGAEA; 10.1594/PANGAEA.962895 (2013).
8. Bartsch, I. et al. Changes in kelp forest biomass and depth distribution in Kongsfjorden, Svalbard, between 1996–1998 and 2012–2014 reflect Arctic warming. *Polar Biol.* **39,** 2021–2036; 10.1007/s00300-015-1870-1 (2016).

9. Schlegel, R. W. et al. Underwater light environment in Arctic fjords. Earth Syst. Sci. Data 16, 2773-2788; 10.5194/essd-16-2773-2024 (2024).

10. Krause-Jensen, D. et al. Seasonal sea ice cover as principal driver of spatial and temporal variation in depth extension and annual production of kelp in Greenland. *Global Change Biol.* **18,** 2981-2994: 10.1111/j.1365-2486.2012.02765.x (2012).

11. Fritsch, F. N. & Carlson, R. E. Monotone piecewise cubic interpolation. *SIAM J. Numer. Anal.* **17**, 238–246; 10.1137/0717021 (1980).

12. Kahaner, D., Moler, C. & Nash, S. Numerical Methods and Software. Upper Saddle River, NJ: Prentice Hall (1989).

13. Kruss, A., Tegowski, J., Tatarek, A., Wiktor, J. & Blondel, P. Spatial distribution of macroalgae along the shores of Kongsfjorden (West Spitsbergen) using acoustic imaging. *Pol. Polar Res.* **38**, 205-229; 10.1515/popore-2017-0009 (2017).

14. Bech, C., Langseth, I., Moe, B., Fyhn, M. & and Gabrielsen, G. W. The energy economy of the arctic-breeding kittiwake (*Rissa tridactyla*): a review. *Comp. Biochem. Phys.* **A 133,** 765-770; 10.1016/S1095-6433(02)00153-8 (2002).

15. Ellis, H. I. & Gabrielsen, G. W. Energetics of free-ranging seabirds in *Marine Birds* (eds. Schreiber, E. A. & Burger, J.), **Chapter 11**, 357-405. CRC Press, Boca Raton, Florida, USA (2002).

16. McNicholl, D. G., Davoran, G. K., Majeswki, A. R. & Reist, J. D. Isotopic niche overlap between co-occurring capelin (*Mallotus villosus*) and polar cod (*Boreogadus saida*) and the effect of lipid extraction on stable isotope ratios. *Polar. Biol.* **41,** 423–432; 10.1007/s00300-017-2199-8 (2018).

17. Vihtakari, M. et al. Black-legged kittiwakes as messengers of Atlantification in the Arctic. *Sci. Rep.-Uk.* **8,** doi:Artn 1178; 10.1038/S41598-017-19118-8 (2018).

18. Torsvik, T. et al. Impact of tidewater glacier retreat on the fjord system: modeling present and future circulation in Kongsfjorden, Svalbard. *Estuar. Coast. Shelf Sci.* **220,** 152-165; 10.1016/j.ecss.2019.02.005 (2019).

19. Sundfjord, A. et al. Effects of glacier runoff and wind on surface layer dynamics and Atlantic Water exchange in Kongsfjorden, Svalbard; a model study. *Estuar. Coast. Shelf Sci.* **187,** 260-272; 10.1016/j.ecss.2017.01.015 (2017).

20. Norwegian Polar Institute. *Terrengmodell Svalbard (S0 Terrengmodell)*; 10.21334/npolar.2014.dce53a47 (2014).

21. Wang, C. N., Chant, R. J. & Jackson, R. H. Parameterizing subglacial discharge in modeling buoyancy driven flow in tidewater glacier fjords. *J. Geophys. Res.-Oceans* **128,** e2023JC019924; 10.1029/2023JC019924 (2023).

22. Jerlov N. G. The optical classification of sea water in the euphotic zone. *Rep. Dept. Phys. Oceanogr.* **36,** Univ. Copenhagen, 46 pp (1978).

23. Gattuso, J. P., Gentili, B., Antoine, D. & Doxaran, D. Global distribution of photosynthetically available radiation on the seafloor. *Earth Syst. Sci. Data* **12,** 1697-1709; 10.5194/essd-12-1697-2020 (2020).

24. Skamarock, W., L. et al. A description of the advanced research WRF version 3, NCAR technical note NCAR/TN-475+STR, p. 113 (2008).

25. Hattermann, T., Isachsen, P. E., von Appen, W. J., Albretsen, J. & Sundfjord, A. Eddy-driven recirculation of Atlantic Water in Fram Strait. *Geophys. Res. Lett.* **43,** 3406-3414; 10.1002/2016GL068323 (2016).

26. Egbert, G. D. & Erofeeva, S. Y. Efficient inverse modelling of barotropic ocean tides, J. *Atmos. Ocean. Tech.* **19,** 183–204; 10.1175/15200426(2002)019<0183:Eimobo>2.0.Co;2 (2002).

27. Taylor, K. E. Summarizing multiple aspects of model performance in a single diagram. *J. Geophys. Res.-Atmos.* **106,** 7183-7192; 10.1029/2000jd900719 (2001).

28. Skogseth, R., Tverberg, V., Walczowski, W. & Sundfjord, A. (2019). Kongsfjorden Transect CTD data 892 1906-2016 [Data set]. Norwegian Polar Institute. https://doi.org/10.21334/npolar.2019.074a215c (2019).

29. Monsen, N. E., Cloern, J. E., Lucas, L. V. & Monismith, S. G. A comment on the use of flushing time, residence time, and age as transport time scales. *Limnol. Oceanogr.* **47,** 1545-1553; 10.4319/lo.2002.47.5.1545 (2002).

30. Lucas, L. V. & Deleersnijder, E. Timescale methods for simplifying, understanding and modeling biophysical and water quality processes in coastal aquatic ecosystems: A review. *Water* **12,** 2717; 10.3390/W12102717 (2020).

31. Edwards, A. & Sharples, F. *Scottish Sea-Lochs - a Catalogue*. Scottish Marine Biological Association, Oban, Scotland (1986). https://pureadmin.uhi.ac.uk/ws/portalfiles/portal/38111606/134_Edwards_A_Sharples_F_Scottish_Sea_Lochs.pdf

32. Haidvogel, D. B. et al. Ocean forecasting in terrain-following coordinates: Formulation and skill assessment of the Regional Ocean Modeling System. *J. Comput. Phys.* **227,** 3595-3624; 10.1016/j.jcp.2007.06.016 (2008).

33. Shchepetkin, A. F. & McWilliams, J. C. The regional oceanic modeling system (ROMS): a split-explicit, free-surface, topography-following-coordinate oceanic model. *Ocean Model.* **9,** 347-404; 10.1016/j.ocemod.2004.08.002 (2005).

34. Shchepetkin, A. F. & McWilliams, J. C. Correction and commentary for "Ocean forecasting in terrain-following coordinates: Formulation and skill assessment of the regional ocean modeling system" by Haidvogel et al., J. Comp. Phys. 227: 3595–3624. *J. Comput. Phys.* **228,** 8985–9000; 10.1016/j.jcp.2009.09.002 (2009).

35. Budgell, W. P. Numerical simulation of ice-ocean variability in the Barents Sea region. *Ocean Dyn.* **55,** 370–387; 10.1007/s10236-005-0008-3 (2005).

36. Röhrs, J. and others. Barents-2.5km v2.0: an operational data-assimilative coupled ocean and sea ice ensemble prediction model for the Barents Sea and Svalbard. *Geosci. Model Dev.* **16,** 5401–5426; 10.5194/gmd-16-5401-2023 (2023).

37. Müller, M., Batrak, Y., Kristiansen, J., Køltzow, M. A. O., Noer, G. & Korosov, A. Characteristics of a convective-scale weather forecasting system for the European Arctic, *Mon. Weather Rev.* **145,** 4771–4787; 10.1175/MWR-D-17-0194.1 (2017).

38. Schmidt, L. S., Schuler, T. V., Thomas, E. E. & Westermann, S. Meltwater runoff and glacier mass balance in the high Arctic: 1991–2022 simulations for Svalbard. *Cryosphere* **17,** 2941–2963. doi: 10.5194/tc-17-2941-2023 (2023).

39. Westermann, S., and others. 2023. The CryoGrid community model (version 1.0) – a multi-physics toolbox for climate-driven simulations in the terrestrial cryosphere. *Geosci. Model Dev.* **16,** 2607–2647; 10.5194/gmd-16-2607-2023 (2023).

40. van Pelt, W. et al. A long-term dataset of climatic mass balance, snow conditions, and runoff in Svalbard (1957-2018). *Cryosphere* **13,** 2259-2280; 10.5194/tc-13-2259-2019 (2019).

41. Calleja, M. L. et al. Effects of increase glacier discharge on phytoplankton bloom dynamics and pelagic geochemistry in a high Arctic fjord. *Prog. Oceanogr.* **159,** 195-210; 10.1016/j.pocean.2017.07.005 (2017).

42. Fransson, A., & Chierici, M. Marine CO_2 system data for the Svalbard fjord Kongsfjorden and the West-Spitsbergen shelf in July 2012-2014 [Data set]. Norwegian Polar Institute; doi.org/10.21334/npolar.2019.e53eae53 (2019).

43. Fransson, A., & Chierici, M. Marine dissolved inorganic carbon (DIC) in Kongsfjorden and shelf, Svalbard, in summer 2015-2017 [Data set]. Norwegian Polar Institute; doi.org/10.21334/npolar.2024.87ca4acd (2024a).

44. Fransson, A. & Chierici, M. Marine dissolved inorganic carbon (DIC) in Kongsfjorden and shelf, Svalbard, in summer 2018-2020 [Data set]. Norwegian Polar Institute; doi.org/10.21334/npolar.2024.dc75c696 (2024b).

45. Wold, A., P. Assmy & Duarte, P. Biogeochemistry data from Kongsfjorden and Rijpfjorden 2011 to 2020 [Data set]. Norwegian Polar Institute; doi.org/10.21334/npolar.2024.4d4de169 (2024).

46. Torres-Valdes, S. et al. FRAM remote access samplers: nutrient and associated sensor data. PANGAEA; doi.org/10.1594/PANGAEA.936749 (2021).

47. Hoppe, C. J. M., Wischnewski, L., Wolf, K. K. E., Cottier, C. F. & Rost, B. Biogeochemical and ecophysiological monitoring at station Kb3 in Kongsfjorden (2014-2018). PANGAEA; doi.org/10.1594/PANGAEA.931854 (2021).

48. van de Poll, W. H. et al. Solar radiation and solar radiation driven cycles in warming and freshwater discharge control seasonal and inter-annual phytoplankton chlorophyll a and

taxonomic composition in a high Arctic fjord (Kongsfjorden, Spitsbergen). *Limnol. Oceanogr*. **66,** 1221–1236; 10.1002/lno.11677 (2021).

49. Cottier, F. et al. Water mass modification in an Arctic fjord through cross-shelf exchange: The seasonal hydrography of Kongsfjorden, Svalbard. *J. Geophys. Res.-Oceans* **110,** Artn C12005; 10.1029/2004jc002757 (2005).

50. Aas, E., Hojerslev, N. K., Hokedal, J. & Sorensen, K. Optical water types of the Nordic Seas and adjacent areas. *Oceanologia* **55,** 471-482; 10.5697/oc.55-2.471 (2013).
